# Primary care detection of Alzheimer’s disease using a self-administered digital cognitive test and blood biomarkers

**DOI:** 10.1038/s41591-025-03965-4

**Published:** 2025-09-15

**Authors:** Pontus Tideman, Linda Karlsson, Olof Strandberg, Susanna Calling, Ruben Smith, Patrik Midlöv, Philip B. Verghese, Joel B. Braunstein, Niklas Mattsson-Carlgren, Erik Stomrud, Sebastian Palmqvist, Oskar Hansson

**Affiliations:** 1 Clinical Memory Research Unit, Department of Clinical Sciences Malmö, Lund University, Lund, Sweden; 2 Memory Clinic, Skåne University Hospital, Malmö, Sweden; 3 Center for Primary Health Care Research, Department of Clinical Sciences Malmö, Lund University, Malmö, Sweden; 4 Office for Primary Care, Skåne University Hospital, Lund, Sweden; 5 C2N Diagnostics LLC, St Louis, MO USA

**Keywords:** Alzheimer's disease, Psychology

## Abstract

After the clinical implementation of amyloid-β-targeting therapies for people with cognitive impairment due to Alzheimer’s disease (AD), there is an urgent need to efficiently identify this patient population in primary care. Therefore, we created a brief and self-administered digital cognitive test battery (BioCog). Based on its sub-scores, a logistic regression model was developed in a secondary care cohort (*n* = 223) and then evaluated in an independent primary care cohort comprising 19 primary care centers (*n* = 403). In primary care, BioCog had an accuracy of 85% when using a single cutoff to define cognitive impairment, which was significantly better than the assessment of primary care physicians (accuracy 73%). The accuracy increased to 90% when using a two-cutoff approach. BioCog had significantly higher accuracy than standard paper-and-pencil tests (that is, Mini-Mental State Examination, Montreal Cognitive Assessment, Mini-Cog) and another digital cognitive test. Furthermore, BioCog combined with a blood test could detect clinical, biomarker-verified AD with an accuracy of 90% (one cutoff), significantly better than standard-of-care (accuracy 70%) or when using the blood test alone (accuracy 80%). In conclusion, this proof-of-concept study shows that a brief, self-administered digital cognitive test battery can detect cognitive impairment and, when combined with a blood test, accurately identify clinical AD in primary care.

## Abstract (web summary)

A brief, self-administered digital cognitive test, in combination with a blood test, accurately detects clinical Alzheimer’s disease in primary care.

## Main

AD is the most common cause of dementia, characterized by depositions of amyloid-β (Aβ), aggregated tau and progressive neurodegeneration^1^. The clinical syndrome of AD typically starts with subjective cognitive decline (SCD), in which individuals report memory problems and/or other cognitive difficulties but perform normally on cognitive tests. This phase is followed by mild cognitive impairment (MCI), marked by objective cognitive symptoms, and later by dementia, characterized by clear functional deficits in activities of daily living^2^. Diagnosing AD is challenging, especially in the early stages of the disease and in primary care. The prevalence of both underdiagnosis and misdiagnosis is high with 20–30% of people being misdiagnosed in specialized care and approximately 40% in primary care when the AD diagnosis is not supported by biomarkers^3–6^. The implementation of biomarker testing such as positron emission tomography (PET) with Aβ aggregate-binding tracer or by lumbar puncture measuring concentrations of Aβ and tau in the cerebrospinal fluid (CSF) has improved diagnostic precision in specialized clinics^7–9^. Nevertheless, these methods are either costly or invasive, and the availability is very limited globally. Recently, blood tests based on tau phosphorylated at threonine 217 (phosphorylated-tau217) have been shown to detect AD pathology with accuracies of around 90% in both primary and secondary care^6^, demonstrating performances comparable to clinically used CSF tests^10^. Therefore, blood tests for AD have the potential to revolutionize the diagnostic work-up of AD in people with cognitive symptoms, especially in primary care centers^9,11^.

The prevalence of AD brain pathology in the population varies by age and apolipoprotein E (*APOE*) status, but even more so by the severity of cognitive symptoms^11^. SCD can arise from a variety of causes, resulting in approximately only 20–25% of individuals with SCD exhibiting AD pathology. By contrast, about 40–60% of individuals with MCI and 70–80% with dementia have AD pathology^12^. As a result, the pretest probabilities vary by cognitive stage, resulting in lower positive predictive values (PPV) of AD biomarkers in people with SCD compared to those with dementia^11,12^. Consequently, a positive AD blood test in an individual with SCD carries a higher risk of being a false-positive than a positive result for an individual with cognitive impairment (MCI or dementia)^6^. The implications of such a false-positive result are substantial, because a person may be incorrectly informed of having AD associated with progressive cognitive decline in the coming years. Furthermore, only individuals with AD with cognitive impairment (but not SCD) are eligible for treatment with the newly implemented Aβ-targeting immunotherapies^1,13–15^. Biomarker testing in clinical practice is currently only recommended in patients with cognitive impairment^1,3,13,16^. This underscores the importance of identifying objective impairment (MCI and dementia) in individuals with cognitive symptoms, which in international guidelines is recommended to always precede AD biomarker testing^5,11,17,18^.

The administration and interpretation of cognitive tests vary substantially among primary care providers, both in and between countries. In addition, primary care providers often lack the time, expertise or resources to conduct assisted, in-clinic, cognitive tests^19^. Brief yet accurate digital cognitive testing, performed either remotely or unsupervised at the primary care center, may offer a viable solution to overcome this gap. We therefore developed a digital, self-administered, cognitive test battery (BioCog) and examined its ability to accurately identify cognitive impairment among people presenting with cognitive symptoms in primary care in a proof-of-concept study. Furthermore, we explored its utility when combined with a blood test to identify individuals with cognitive impairment due to AD.

## Results

### Study participants

We included 223 participants with cognitive symptoms from a secondary care cohort: the ongoing BioFINDER-2 study (NCT03174938) (Table 1)^20^. The mean (s.d.) age was 73 (8.8) years, 128 (57%) were male and 119 (53%) had objectively verified cognitive impairment. In addition, 403 participants were included from a primary care cohort, BioFINDER-Primary Care study (NCT06120361) (Table 1 and Supplementary Fig. 1), which recruits people seeking help for cognitive symptoms at 19 primary care units^6^. Only people for whom the primary care physician (PCP) thought neurodegenerative disease was a reasonably possible cause of the symptomatology were invited to participate in the study. The mean (s.d.) age of the primary care cohort was 77 (8.0) years, 194 (48%) were male and 229 (57%) had objectively verified cognitive impairment.

**Table 1:** Characteristics of the primary and secondary care cohorts

	Care cohort		Standardized between-group difference	
	Primary	Secondary	Median (95% CI)^a^	Percent (95% CI)^b^
Number of participants	403	223		
Age median (IQR), years	77 (72 to 82)	73 (66 to 78)	0.52 (0.26 to 0.81)	
Sex, *n* (%)				
Female	209 (52)	95 (43)		2.2 (0.33 to 4.1)
Male	194 (48)	128 (57)		−2.2 (−4.1 to −0.35)
Length of education median (IQR), years	11 (9 to 13)	13 (11 to 16)	−0.60 (−0.92 to −0.30)	
Carrier of *APOE* ε4, *n*; total (%)	169; 402 (42)	130; 220 (59)		−4.1 (−6.1 to −2.2)
Mini-Mental State Examination				
Number of participants	402	223		
Score, median (IQR)	27 (25 to 29)	27 (24 to 29)	−0.14 (−0.32 to 0)	
Cognitive impairment (RBANS < 78), *n* (%)^c^				
Negative	174 (43)	104 (47)		−0.83 (−2.7 to 1.1)
Positive without dementia	124 (31)	66 (30)		0.31 (−1.6 to 2.4)
Positive with dementia	105 (26)	53 (24)		0.64 (−1.4 to 2.6)
Aβ status^d^, *n*; total (%)				
Negative	196; 403 (49)	84; 222 (38)		2.6 (0.8 to 4.8)
Positive	207; 403 (51)	138; 222 (62)		−2.6 (−4.8 to −0.7)
Medical history, *n* (%)				
Cardiovascular disease	303 (75)	120 (54)		5.4 (3.4 to 7.5)
Hyperlipidemia	249 (62)	110 (49)		3.0 (0.87 to 5.0)
Chronic kidney disease	108 (27)	46 (21)		1.8 (−0.20 to 3.8)
Diabetes	78 (19)	34 (15)		1.3 (−0.59 to 3.4)

^a^Standard median difference (difference between medians divided by pooled s.d.).

^b^Standardized difference in proportions.

^c^Proxy variable based on similar paper-and-pencil tests for secondary care cohort (see details in Supplementary Methods).

^d^Biomarker confirmed, based on either CSF Aβ42/Aβ40 or Aβ-PET.

Compared with secondary care, participants in the primary care cohort were older, had fewer years of education, a higher prevalence of cardiovascular disease and hyperlipidemia, and a larger proportion were female. A slightly smaller proportion of participants in primary care were Aβ-positive and *APOE* ε4 carriers. Similar proportions were cognitively impaired (Table 1).

All participants performed the self-administered digital test battery, BioCog, comprising: (1) a word list test with immediate recall, delayed recall and recognition of ten words; (2) a cognitive processing speed task; and (3) questions about orientation to time (Supplementary Fig. 2). The study’s primary outcome was objectively verified cognitive impairment determined on the basis of a standard battery of cognitive tests, the Repeatable Battery for the Assessment of Neuropsychological Status (RBANS), administered by neuropsychologists at a memory clinic. The secondary care cohort was used for training to establish cognitive test models and cutoffs. The primary care cohort was used for validation where the models and cutoffs were applied.

### Psychometric properties of BioCog

The subtests of BioCog demonstrated moderate negative Spearman’s correlations with age (−0.31 to −0.58), and weak positive correlations with education (0.13 to 0.33). Performance across subtests did not differ by sex in either cohort, except for immediate recall and recognition in the primary care cohort, where female participants performed slightly better (Supplementary Tables 1 and 2). Strong correlations (0.57 to 0.83) were observed between BioCog subtests and their corresponding paper-and-pencil analogs (for example, BioCog processing speed test and Symbol Digit Modalities Test), supporting convergent validity. Correlations between individual BioCog subtests and a short-term memory task were weak (0.11 to 0.29). Similarly, only weak correlations were observed between BioCog subtests and a measure of visuoperceptual or spatial functioning (0.13 to 0.27), indicative of divergent validity (Supplementary Tables 3 and 4). Participants with cognitive impairment scored significantly lower on all BioCog measures compared to cognitively unimpaired individuals (Supplementary Tables 5 and 6). Internal consistency of the subtests, estimated by McDonald’s omega (*ω*), a reliability coefficient, ranged from acceptable to excellent (0.70 to 0.90) (Supplementary Table 7). Self-reported difficulty in understanding test instructions was very low (2%), and most participants reported that the tasks were engaging or were neutral (word list test 80%; processing speed test 91%) (Supplementary Fig. 3). Average completion time for BioCog was 11.2 min (s.d. 1.02 min), similarly across both cognitively unimpaired and cognitively impaired participants (Supplementary Table 8). Overall, these results suggest that BioCog is a valid, reliable and feasible tool for assessing cognitive function.

### Establishing a BioCog model in the secondary care cohort

In the secondary care cohort, multiple logistic regression models that could predict the presence or absence of cognitive impairment were established using basic demographics (three variables: age, sex and education level) and BioCog (six variables from the subtests), in total nine variables (Supplementary Table 9). When creating models, recursive variable selection was performed to decrease the risk of overfitting. The best model based on the Akaike information criterion (AIC) was selected. The model with lowest AIC included six variables (BioCog_6_): delayed ten-word recall (number of correct answers), cognitive processing speed task (number of correct answers), three repetitions of immediate ten-word recall (number of correct answers), age, delayed ten-word recognition (number of correct answers) and three repetitions of immediate ten-word recall (total time); listed in the order of feature importance.

**Fig. 1:**
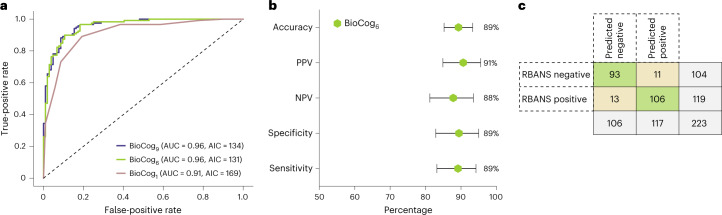
Establishing and evaluating models in the secondary care cohort. Model performances in the secondary care cohort (which was also used to fit the models and to establish the cutoff), *n* = 223. **a**, ROC curves, AUCs and AICs for the three BioCog models using one (BioCog_1_), six (BioCog_6_) or nine (BioCog_9_) input variables. **b**, Evaluation of BioCog_6_ selecting an optimal cutoff point based on the maximum value of Youden’s index (probability = 0.575). Error bars indicate 95% CI, with the center point corresponding to the mean value. **c**, Cross-tabulation analysis using BioCog_6_.

For comparison, receiver operating characteristic (ROC) curves from BioCog_6_ (area under the curve (AUC) = 0.96) together with models containing all variables (BioCog_9_, AUC = 0.96, in addition including orientation to time (correct answers), education level and sex), or only the best performing single subtest (BioCog_1_, AUC = 0.91, only delayed ten-word recall), are provided in Fig. 1a. In the secondary care cohort, BioCog_6_ demonstrated an accuracy of 89% (95% confidence interval (CI) 84–93%), PPV of 91% (95% CI 85–95%), negative predictive value (NPV) of 88% (95% CI 81–93%), specificity of 89% (95% CI 83–95%) and sensitivity of 89% (95% CI 83–94%) for detection of cognitive impairment (Fig. 1b). Comparisons of true versus predicted classes are shown in a cross-tabulation analysis (Fig. 1c).

**Extended Data Fig. 1:**
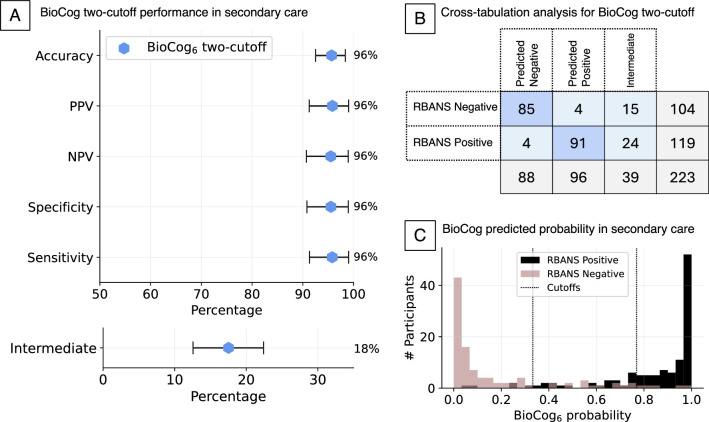
Evaluating a two-cutoff approach for the BioCog_6_ model in the secondary care cohort. BioCog_6_ two-cutoff model performance in the secondary care cohort (which was also used to fit the model and to establish the cutoffs). The two cutoffs were set at probabilities 0.332 and 0.769, corresponding to 95% sensitivity and 95% specificity respectively. **a**) Evaluation of the BioCog_6_ two-cutoff model. Error bars indicate 95% CIs, with the centre point corresponding to the mean value. **b**) Cross-tabulation analysis the BioCog_6_ two-cutoff model. **c**) Histogram of the predicted probabilities from the BioCog_6_ model (x-axis) colored by the true binary RBANS proxy composite score (outcome variable).

To enhance diagnostic performance, a two-cutoff approach (as previously proposed^3,6,9,21–23^) was applied using BioCog_6_. This way, participants with predicted probabilities close to the one-cutoff decision boundary formed an intermediate group and were not considered when evaluating performance (representing individuals who would need further evaluation to determine their cognitive status). The two-cutoff probabilities of 0.332 and 0.769 were chosen to achieve 95% sensitivity and 95% specificity, respectively. Using these cutoffs, BioCog_6_ had an accuracy of 96% (95% CI 92–98%), PPV of 96% (95% CI 91–99%), NPV of 96% (95% CI 90–98%), specificity of 96% (95% CI 90–98%) and sensitivity of 96% (95% CI 91–99%) (Extended Data Fig. 1). The intermediate group comprised 18% of the cohort (95% CI 12–22%).

### Validating the BioCog model in the primary care cohort

**Fig. 2:**
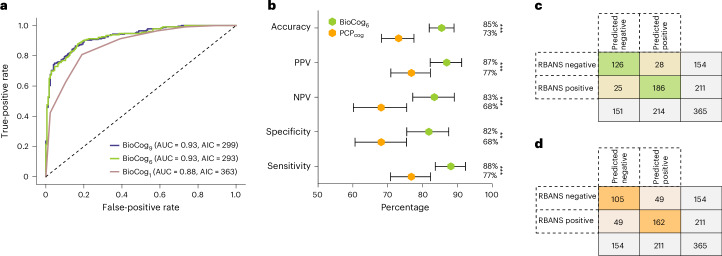
Evaluating models in the primary care cohort. Model performances in the primary care cohort (which was not used to fit the models or to establish the cutoff). **a**, ROC curves, AUCs and AICs for the three BioCog models using one (BioCog_1_), six (BioCog_6_) or nine (BioCog_9_) input variables, *n* = 403. **b**, Evaluation of BioCog_6_ based on the optimal cutoff point established in the secondary care cohort (probability = 0.575), *n* = 365. Compared to a PCP diagnosis of SCD versus MCI or dementia (PCP_cog_). Error bars indicate 95% CI, with the center point corresponding to the mean value. CI and two-sided *P* values were computed using bootstrapping (*n* = 5,000 resamples with replacement) and adjusted for multiple comparisons (Benjamini–Hochberg). **c**, Cross-tabulation analysis using BioCog_6_. **d**, Cross-tabulation analysis using PCP_cog_ diagnoses. Comparisons in **b**–**d** were made on a subset of individuals with existing PCP_cog_ evaluation data (*n* = 365). **P < 0.01, ***P < 0.001 (assessed with bootstrapping and false discovery rate (FDR) corrected). Exact *P* values in **b**: *P*_Accuracy_ = 0.0007, *P*_PPV_ = 0.001, *P*_NPV_ = 0.0007, *P*_Specificity_ = 0.005, *P*_Sensitivity_ = 0.0007. Nominal significance <0.0001 was set to 0.0001 during FDR correction, yielding a lowest adjusted *P* value of 0.0007.

Next, we evaluated the predictive ability of the previously established BioCog models and cutoffs in the independent primary care cohort. BioCog_6_ achieved higher or similar AUC (0.93) compared to BioCog_1_ (AUC = 0.88) and BioCog_9_ (AUC = 0.93) when identifying cognitive impairment (Fig. 2a). In the primary care cohort, BioCog_6_ had an accuracy of 85% (95% CI 81–89%), PPV of 87% (95% CI 82–91%), NPV of 83% (95% CI 77–89%), specificity of 82% (95% CI 75–87%) and sensitivity of 88% (95% CI 83–92%) (Fig. 2b). Of the 96 cognitively impaired participants without AD, 82 were correctly identified as impaired by the BioCog_6_ model (85% accuracy), suggesting similar performance in this subpopulation. The BioCog_6_ model was compared to standard assessment of PCPs (PCP_cog_), which included brief cognitive testing with Mini-Mental State Examination (MMSE) and The Montreal Cognitive Assessment (MoCA), computed tomography scan of the brain and a clinical assessment of the participant. BioCog_6_ had significantly higher performance across all evaluation metrics (Fig. 2b) (accuracy BioCog_6_ = 85% versus PCP = 73%; *P* < 0.001). Comparisons of true versus predicted classes for BioCog_6_ and PCP_cog_ assessments can be seen in cross-tabulation analyses (Fig. 2c,d).

**Fig. 3:**
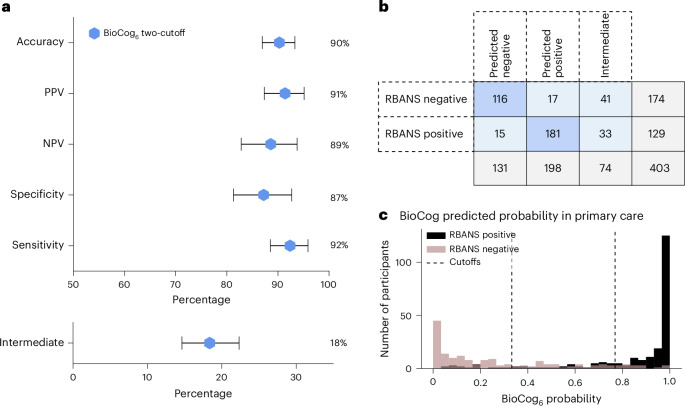
Evaluating a two-cutoff approach for the BioCog_6_ model in the primary care cohort. BioCog_6_ two-cutoff model performance in the primary care cohort (which was not used to fit the model nor to establish the cutoffs), *n* = 403. **a**, Evaluation of the BioCog_6_ two-cutoff model (*n* = 329, without the participants in the intermediate group). Error bars indicate 95% CI, with the center point corresponding to the mean value. **b**, Cross-tabulation analysis using the BioCog_6_ two-cutoff model. **c**, Histogram of the predicted probabilities from the BioCog_6_ model (*x* axis) colored by the true binary RBANS composite score (outcome variable).

Using two cutoffs (applying the cutoffs previously established in the secondary care cohort), BioCog_6_ showed an accuracy of 90% (95% CI 86–93%), PPV of 91% (95% CI 87–95%), NPV of 89% (95% CI 82–93%), specificity of 87% (95% CI 81–92%) and sensitivity of 92% (95% CI 88–95%) (Fig. 3). The intermediate group comprised 18% of the participants (95% CI 14–22%).

Next, a head-to-head comparison was performed between the BioCog_6_ model and several widely used standard paper-and-pencil tests (MMSE, MoCA, Mini-Cog) and another digital test (Cambridge Neuropsychological Test Automated Battery (CANTAB)). Using one cutoff, the BioCog_6_ model significantly outperformed all the other cognitive tests on accuracy (Table 2). This result remained consistent when cognitive tests were treated as continuous variables and adjusted for demographics (Supplementary Fig. 4). It also remained when removing age as an input in the BioCog_6_ model (Supplementary Table 10). For the two-cutoff approach, BioCog_6_ demonstrated significantly higher accuracy than MoCA, the only other test adapted for a two-cutoff approach (90% versus 76%, *P* = 0.001), and with a smaller, but not significantly different intermediate group size (18% versus 23%, *P* = 0.07).

**Table 2:** Head-to-head comparison between BioCog_6_ and other cognitive tests when predicting the binary RBANS composite in the primary care cohort

	Cutoff(s) for positivity	Accuracy (95% CI); FDR-corrected *P* value compared to BioCog_6_	PPV (95% CI); FDR-corrected *P* value compared to BioCog_6_	NPV (95% CI); FDR-corrected *P* value compared to BioCog_6_	Specificity (95% CI); FDR-corrected *P* value compared to BioCog_6_	Sensitivity (95% CI); FDR-corrected *P* value compared to BioCog_6_	Intermediate (95% CI); FDR-corrected *P* value compared to BioCog_6_
One-cutoff approaches							
BioCog_6_	>0.575	84% (81–88%)	84% (79–89%)	84% (79–90%)	79% (73–85%)	88% (84–92%)	–
MMSE	<27	71% (67–76%); 0.0007	81% (75–87%); 0.30	64% (58–70%); 0.0007	80% (75–86%); 0.70	64% (58–71%); 0.0007	–
MoCA	<26	67% (62–71%); 0.0007	63% (58–68%); 0.0007	93% (86–100%); 0.06	27% (20–34%); 0.0007	98% (96–100%); 0.0007	–
Mini-Cog	<4	75% (71–79%); 0.0007	78% (72–83%); 0.02	71% (64–78%); 0.0007	72% (65–79%); 0.08	77% (71–83%); 0.0007	–
CANTAB	>41	76% (71–80%); 0.0007	78% (73–83%); 0.03	73% (66–79%); 0.001	72% (65–79%); 0.08	78% (73–84%); 0.001	–
Two-cutoff approaches							
BioCog_6_	>0.769, positive <0.332, negative	90% (86–93%)	90% (86–94%)	89% (83–94%)	86% (80–92%)	92% (88–96%)	18% (14–22%)
MoCA	<24, positive >26, negative	76% (71–81%, 0.001)	74% (68–79%, 0.001)	90% (80–100%, 0.8)	31% (22–40%, 0.001)	98% (96–100%, 0.008)	24% (20–28%, 0.07)

Diagnostic performance (accuracy, PPV, NPV, sensitivity and specificity) was calculated using the BioCog_6_ model (established in the secondary care cohort) or prespecified cutoffs from the literature. Comparisons were made on a subset of individuals with existing data for all cognitive tests (*n* = 381). Confidence intervals and two-sided *P* values were computed using bootstrapping (*n* = 5,000 resamples with replacement) and adjusted for multiple comparisons (Benjamini–Hochberg). Nominal significance <0.0001 was set to 0.0001 during FDR correction, yielding a lowest adjusted *P* value of 0.0007.

### Digital test and blood biomarkers compared to standard care

**Fig. 4:**
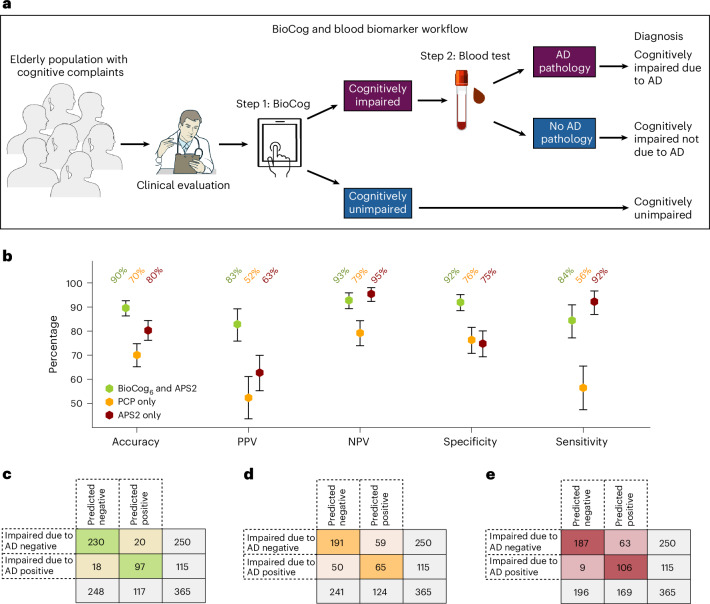
Comparing a digital testing and blood biomarker-based diagnostic workflow to the current standard clinical evaluation in the primary care cohort. Comparisons were made on a subset of individuals with all existing data available (*n* = 365; see Supplementary Table 17 for detailed population characteristics). **a**, Our proposed primary care two-step workflow consisting of step 1, detection of cognitive impairment using the BioCog, followed by step 2 in cognitively impaired individuals, a blood biomarker assessment to evaluate whether AD pathology is present. **b**, Evaluation of the workflow using our BioCog_6_ model for step 1 and the plasma biomarker APS2 for step 2 (green). The workflow was compared against a standard clinical evaluation by PCPs in which the physician assesses both whether the patient had cognitive impairment (MCI or dementia) and whether the impairment was caused by AD (without any biomarkers) (PCP_AD_, orange), and against a workflow using only the plasma biomarker APS2 without any cognitive assessment (red). Error bars indicate 95% CI, with the center point corresponding to the mean value. Significance comparisons between all workflows are given in Supplementary Table 11. **c**–**e**, Corresponding cross-tabulation analyses for BioCog and blood marker workflow (**c**), PCP workflow (**d**) and blood biomarker only workflow (**e**). Credit: Images in **a** adapted from NIAID NIH BIOART (https://bioart.niaid.nih.gov).

Finally, we simulated a two-step diagnostic workflow in primary care that can be performed in addition to the standard clinical evaluation made by a physician to identify biomarker-verified clinical (symptomatic) AD: step 1, detection of objective cognitive impairment using BioCog (BioCog_6_); and step 2, blood biomarker testing (PrecivityAD2; Amyloid Probability Score-2 (APS2)) in those who are cognitively impaired according to BioCog (Fig. 4a). We compared this workflow in the primary care cohort against current standard clinical evaluation by PCPs, where the physician first assessed whether the patient had cognitive impairment and second, whether the impairment was caused by AD (PCP_AD_).

When cognitive impairment (based on neuropsychological assessment) and a clinical AD diagnosis (based on a consensus diagnosis done by dementia experts including CSF analysis or Aβ-PET assessments) was used as outcome, the two-step diagnostic workflow of BioCog (one cutoff) and blood biomarker achieved an overall clinical AD diagnostic accuracy of 90% (95% CI 86–92%), PPV of 83% (95% CI 75–89%), NPV of 93% (95% CI 89–95%), specificity of 92% (95% CI 88–95%) and sensitivity of 84% (95% CI 77–90%) (Fig. 4b). By contrast, PCPs had an overall accuracy of 70% (95% CI 65–74%), PPV of 52% (95% CI 43–61%), NPV of 79% (95% CI 73–84%), specificity of 76% (95% CI 71–81%) and sensitivity of 56% (95% CI 47–66%), all significantly lower than the new digital test and blood biomarker workflow (Fig. 4b).

In comparison, relying solely on the blood biomarker to identify clinical AD without using BioCog maintained a high sensitivity of 92% (95% CI 86–96%) and NPV of 95% (95% CI 92–98%), but showed significantly lower accuracy of 80% (95% CI 76–84%), PPV of 63% (95% CI 55–69%) and specificity of 75% (95% CI 69–80%) compared to the combined BioCog and blood biomarker workflow (Fig. 4b and Supplementary Table 11). Notably, a high proportion (17%) of individuals were classified as false positives when only using the blood test (Fig. 4e). Using the BioCog_6_ and APS2 workflow resulted in significantly higher accuracy than combining APS2 with the traditional cognitive assessments MMSE and MoCA (Supplementary Table 12).

If using a two-cutoff approach for both BioCog and the blood biomarker, the accuracy increased to 95% (95% CI 91–97%), with 30% (95% CI 25–34%) of individuals in the intermediate group (Supplementary Fig. 5). All results were similar when using cognitive impairment and an abnormal Aβ CSF or PET biomarker as outcome instead of a clinical AD diagnosis (Supplementary Fig. 5), and when applying the clinically used APS2 cutoff (Supplementary Fig. 6).

### Sensitivity analyses

A Clinical Dementia Rating (CDR) global score of ≥0.5 was evaluated as the reference standard for cognitive impairment instead of RBANS. When directly applying the BioCog_6_ model in the primary care cohort using this outcome, the performance remained high (Supplementary Fig. 7 and Supplementary Table 13). The AUC decreased from 0.93 to 0.90, accuracy from 85% to 82%, and PPV from 87% to 86%. These results highlight the robustness and generalizability of the BioCog_6_ model in predicting cognitive impairment, also when using a different reference standard.

Additional evaluation metrics for the main analyses in the primary care cohort (including balanced accuracy, harmonic mean of precision and recall (F1 score), and positive and negative likelihood ratios) (Supplementary Tables 14 and 15) supported our main findings.

## Discussion

This study demonstrated that a brief digital cognitive assessment battery detects cognitive impairment with an accuracy of 85% when validated in an elderly primary care population presenting with cognitive symptoms. The accuracy of the self-administered BioCog test was significantly higher than that of standard-of-care methods, including paper-and-pencil tests MMSE, MoCA and Mini-Cog, which require the presence of healthcare personnel when administered. The digital test on its own also outperformed clinical evaluation by the PCP, which included patient history and standard cognitive tests. Notably, higher performance was consistent whether cognitive impairment was defined using RBANS or CDR, underscoring BioCog’s robustness and generalizability in detecting objective cognitive impairment. Furthermore, in a two-step workflow combining BioCog with a blood biomarker test, clinical AD (that is, cognitive impairment due to AD) could be identified with significantly higher accuracy than the standard PCP assessment (90% versus 70%), or using a blood biomarker test without previous digital cognitive assessment (90% versus 80%). Beyond high accuracy, digital tests like BioCog offer distinct advantages over paper-and-pencil assessments. BioCog enables time-efficient cognitive evaluations and eliminates variability introduced by differences in scoring or interpretation, which are critical features in primary care^19^. Furthermore, it facilitates the simultaneous collection of different variable categories, such as time, alongside correct answers, allowing for more comprehensive data. Digital platforms also make it easier to utilize advanced statistical models, moving beyond simple threshold-based scoring of sums (used in MMSE, MoCA and Mini-Cog) to leverage more nuanced, data-driven insights without adding significant processing time or requiring advanced interpretation skills.

Compared to another digital test that has been CE marked and US Food and Drug Administration approved and is currently used in clinical practice for dementia evaluations (CANTAB), BioCog demonstrated significantly higher accuracy in identifying cognitive impairment (85% versus 76%) (Table 2). Our study did not evaluate all available digital tablet solutions, such as Cognivue^24^, NIH Toolbox^25^, cCOG^26^, Cogstate^27^ or TabCAT^28^, considering the load this would impose on participants, and to avoid learning biases. To our knowledge, none of these digital tests have been evaluated in primary care. Future research comparing various brief and self-administered digital tools in primary care will be essential to ensure that clinicians have access to the most effective options for detecting cognitive impairment. Importantly, a central conclusion of this study is that combining digital cognitive testing and a blood biomarker can enhance not only the efficiency of the diagnostic process in primary care, but also the diagnostic certainty of AD.

A diagnostic workflow (Fig. 4) combining a positive BioCog assessment with an accurate blood biomarker test offers an efficient and reliable way to diagnose clinical AD with high certainty (accuracy of 90% compared to 70% for current standard-of-care without biomarkers and 80% for blood biomarkers only). These results suggest that digital cognitive tests, when combined with blood biomarker tests, have potential to significantly enhance the clinical work-up of AD and can become highly valuable to efficiently align clinical AD diagnostics with global recommendations. The World Health Organization’s preferred product profile for AD blood tests emphasizes that such tests should be used exclusively in individuals with cognitive impairment^17^, where the pretest probability is high enough to achieve a PPV of >90% without the need for confirmatory CSF or PET biomarkers. This aligns with other international recommendations^11,16^. In addition, only individuals with cognitive impairment are eligible for treatment with acetylcholinesterase inhibitors or recently developed Aβ-targeting immunotherapies^1,13–15^. This also underscores the importance of identifying cognitive impairment in primary care with efficient and accurate tools like BioCog, to enable referral of as many eligible patients as possible to specialist clinics for treatment with Aβ immunotherapies without substantial burden on the primary care system. Further evaluation (for example magnetic resonance imaging scans, *APOE*, contraindicated medications) of potential treatment candidates is required at specialty clinics before initiating treatment^29^.

For digital assessments and blood tests to be effective in diagnosing clinical AD, it is essential that they demonstrate very high PPVs, particularly before initiating expensive treatments. In addition, it is of high importance that their predictive properties are robust, with strong generalization to new patients. Blood biomarker tests have shown high performance across many secondary care cohorts^9,30^, as well as in primary care^11^, but their PPV varies by age and cognitive status because of differences in pretest probabilities^31^, so their use should be adapted to the clinical context. In this study, using BioCog to assess cognitive impairment expectedly showed a slight decrease in performance between the secondary care cohort (used to fit the model, AUC = 0.96 and PPV = 91%) and the more clinically diverse primary care cohort (only used for model evaluation, AUC = 0.93 and PPV = 87%). To develop a robust BioCog model, we applied recursive feature selection with AIC penalization and focused on external model validation in the independent primary care cohort.

We also investigated the ability of BioCog_6_ to identify cognitive impairment with CDR as outcome instead of RBANS. Whereas RBANS offers a more objective measure of cognitive impairment, CDR captures functional decline^32,33^. Both are widely used in clinical trials and routine clinical practice^14,15,34,35^. When using a CDR global score ≥0.5 as outcome, performance remained high, with only a slight decrease (for example, accuracy from 85% to 82%, and PPV from 87% to 86%). Because the BioCog_6_ model was optimized to detect cognitive impairment (RBANS) and not functional impairment (CDR), a drop in performance is expected. Nevertheless, this decrease was minimal, and BioCog_6_ still outperformed both the clinical judgment of PCPs and other cognitive tests. To further increase diagnostic performance of the BioCog_6_ model, we applied a two-cutoff approach, which resulted in a PPV that remained >90% even in primary care and with 18% of individuals in the intermediate group. These results are encouraging, with BioCog demonstrating a high level of robustness and adaptability, even when applied to a cohort with differing demographic characteristics and a higher prevalence of comorbidities (Table 1), and when defining cognitive impairment using a different reference standard. For the two-cutoff approach, people with intermediate results would need more comprehensive cognitive evaluations. Alternatively, repeated cognitive testing incorporating changes in test scores may help identify cognitive impairment in those individuals^36^.

The findings of our study highlight the key role digital cognitive tools can play in optimizing diagnostic workflows, particularly when combined with blood biomarker tests for a more precise evaluation of clinical AD. Beyond individual test performance, the broader impact of digital assessments lies in their ability to enhance efficiency, standardization and scalability in cognitive assessments^37^. Importantly, their greatest value emerges when integrated into a broader clinical evaluation, in which results can be interpreted alongside other clinical information to support well-informed diagnostic decisions and strengthen the healthcare professional’s confidence in making a correct diagnosis^38^. Even though this study focuses on highlighting the clinical value of BioCog by showing its independent contribution in identifying objective cognitive impairment and clinical AD, our proposal is not to use BioCog or other digital tests as stand-alone tools. BioCog is aimed to complement, not replace, clinical judgment, and further validation in additional research studies and integration into structured care pathways is essential before deployment. In addition, future implementation-based research in different healthcare systems is needed to evaluate the impact on clinical management and to establish clinical practice guidelines before digital assessments combined with blood biomarkers can be used as part of everyday primary care in real-world settings.

There are limitations to this study. First, we established the BioCog model in a secondary care cohort, which differed on demographic factors like age and education level, and on comorbidity prevalence compared to the primary care cohort, representative of the intended use population. However, the robust results across cohorts despite these differences also highlight the generalizability of the model and cutoffs. Second, although BioCog performed well in identifying cognitive impairment, further research is needed to evaluate its utility in longitudinal settings and its ability to monitor disease progression. Third, BioCog was developed and evaluated in Swedish cohorts. It has been translated into several languages, including English, Finnish, Dutch and Spanish, and validation in these languages and in more diverse populations is important to further assess the generalizability. Such validation will be crucial before clinical implementation. Notably, paper-and-pencil versions of similar types of tests have performed very well across different languages and cultures. Fourth, this study did not directly assess how the use of a digital tool influences the clinical work-up in primary care, or whether physicians find it beneficial in practice. These questions will be addressed in future work. Fifth, BioCog was not evaluated as a screening tool for the general population. Instead, it was assessed in individuals who presented with cognitive complaints in primary care, where a physician considered a neurodegenerative disease to be a possible cause. People whose symptoms were clearly attributable to other causes (for example, depression or sleep disorders) were treated or referred accordingly and were not included in the study. This limits our ability to assess BioCog’s utility for screening of the general population. However, such screening currently lacks evidence for improving decision-making or other important outcomes^39^. We therefore argue that the current study, limited to participants with cognitive symptoms possibly caused by an underlying neurodegenerative disease, is clinically more relevant and reflects a realistic scenario for how digital cognitive tools may be integrated to support physicians in primary care.

In summary, this proof-of-concept study demonstrates that a self-administered digital test is effective for identifying objective cognitive impairment in a population with cognitive symptoms for whom the PCP thought a neurodegenerative disease was a possible cause of the symptomatology. Furthermore, when combined with a blood biomarker test, the digital cognitive test could reliably diagnose clinical AD with high accuracy, outperforming the current standard-of-care in primary care. These findings have the potential to transform clinical diagnostics of AD in primary care, enabling detection and treatment in a resource efficient manner.

## Methods

### Participants

All participants provided written informed consent before enrollment in the study, and ethical approval for the study was obtained from the Swedish Ethical Review Authority. Participants from two cohorts were included, one from secondary care and one from primary care. Data for this study were collected between February 2022 and December 2024. The secondary care cohort was used as the training cohort to establish cognitive test models and cutoffs. The primary care cohort was used as the validation cohort were the models and cutoffs were applied.

### Secondary care

Secondary care participants were recruited from the ongoing BioFINDER-2 study (NCT03174938), described in detail elsewhere^20^. Briefly, the study consecutively enrolls participants at the secondary care memory clinic of Skåne University Hospital and the memory clinic of Ängelholm Hospital, and includes a diverse population of study participants. For the current study, patients with either SCD, MCI or dementia were included. Further details are given in the Supplementary Methods.

### Primary care

The BioFINDER-Primary Care study (NCT06120361), described previously^6^, is an ongoing study that recruits patients from primary care centers in southern Sweden. The study consecutively includes patients seeking medical help due to cognitive symptoms either self-reported or reported by a close relative or spouse. In addition, inclusion may also be initiated based on the PCP’s suspicion of a neurocognitive disorder. To ensure a realistic and ethical study design aligned with standard care, the primary care cohort included only individuals for whom the treating PCPs, after the first assessment, considered a potential neurodegenerative disease. People whose symptoms were assessed to be clearly attributable to other causes (for example, depression, sleep disorders) were not enrolled and instead treated at the primary center or referred for appropriate care elsewhere. Patients already diagnosed with dementia or suffering from an unstable severe systemic illness as well as those with ongoing significant alcohol or substance abuse were excluded. At their respective primary care center, patients underwent standard clinical evaluation (‘standard-of-care’), including medical examination by the PCP, cognitive testing, standard blood assessments to rule out other causes and structural brain imaging. Based on the assessments of PCPs, only 29.1% (106 of 364) of the participants would have been referred to secondary care if not included in this study, underscoring that the primary and secondary care cohorts represented different patient populations. For this study patients from 19 primary care centers were included. Further details of the cohort, cognitive testing and inclusion and exclusion criteria are provided in Supplementary Methods.

### The digital cognitive battery (BioCog)

The digital cognitive battery, BioCog, was developed using Python and Kivy to run on an Android tablet. The battery was designed to include a primary part and an optional secondary part that contained more demanding tasks (and was therefore not used in this study). During development of the battery, it was tested by cognitively unimpaired volunteers to improve the user experience. Following this pilot phase, participants in the BioFINDER-Primary Care study completed the battery and provided feedback on how they perceived the tasks and instruction comprehensibility. After additional adjustments, digital testing was introduced in the study design and data collection started. None of the participants in this study had previous experience with the BioCog test battery. The first part of the self-administered test battery, which was used in the current study, had an average completion time of 11.2 min, and is comprised of a word list test with immediate and delayed recall as well as a delayed recognition part, a cognitive processing speed task and questions about orientation to the weekday, date, month and year.

### Comparison between the digital test battery and other cognitive tests

To evaluate the performance of the digital test to detect cognitive impairment, it was compared to paper-and-pencil tests frequently used in primary care settings (which require healthcare personal to perform the tests) and to a well-known, CE marked and US Food and Drug Administration approved digital test. The brief paper-and-pencil tests used, were: (1) the MMSE^40^, the world’s most used cognitive test, which consists of several shorter tasks and has a maximum score of 30 points; (2) MoCA^41^, a widely used screening tool with a maximum of 30 points, which evaluates a broader range of cognitive domains than the MMSE; (3), Mini-Cog^42^, which was developed as a very brief screening test that includes a three-word delayed recall task and clock drawing and has a maximum of 5 points; and (4) the digital, tablet-based paired associates learning task (PAL)^43^ from the CANTAB, which measures visual associative memory. For the main analyses, externally established and validated cutoffs were used for all measures: MMSE <27 points^44^, MoCA <26 points^41^, Mini-Cog <4 points^45^ and for PAL >41 adjusted errors in total^46^. For MoCA a two-cutoff approach was also included using <24 points as the lower cutoff and 26 as the upper cutoff^21^. For the other tests no validated two-cutoff approaches were available. As sensitivity analyses, the cognitive tests were treated as continuous variables and adjusted for demographics.

### PCP estimation of cognitive stage and etiology

The standard clinical evaluation at the primary care units included brief cognitive testing (MMSE and MoCA), computed tomography scan of the brain, and a clinical assessment of the patient. After completing this evaluation, PCPs were asked to report the cognitive stage (SCD, MCI or dementia) and if assessed as MCI or dementia, the most likely cause of the cognitive impairment. The latter was binarized as AD or non-AD. Mixed etiologies including AD (for example, AD with vascular co-pathology) were coded as AD.

### Plasma sampling and analysis

Blood was collected in EDTA tubes and centrifuged within 1 h. Plasma was then pipetted to 1-ml LoBind tubes and shipped the same day to be frozen at −80 °C. Plasma samples were then shipped to and analyzed by C2N Diagnostics in the United States continuously during the study period (47 occasions). Mass spectrometry assays were used to analyze Aβ42, Aβ40, phosphorylated-tau217 and nonphosphorylated-tau217, as previously described^6^. These biomarkers were combined in a logistic regression model (PrecivityAD2 test) to yield a score from 0 to 100 called the APS2 (ref. ^47^). An APS2 cutoff value of 36 was used, previously defined in a clinical practice research study established at 90% specificity for AD pathology^6^. In addition, a two-cutoff approach with values corresponding to 95% sensitivity and 95% specificity (31 lower, 62 upper) was used, also previously defined^6^.

### Outcomes

The primary outcome was objectively verified cognitive impairment. In the primary care cohort, this was established using the RBANS^48^, administered by neuropsychologists at the memory clinic of Skåne University Hospital. The RBANS is a widely used cognitive test battery that has shown high accuracy in detecting and characterizing cognitive deficits associated with AD^49^ and MCI in general^50^. The test battery comprises 12 subtests assessing 5 cognitive domains: immediate and delayed memory, visuospatial and visuoconstructional ability, verbal ability and attention, and requires about 30 min to perform. Using normative data from the Swedish manual generated an age-corrected standard (*M* = 100, s.d. = 15) total scale score for each participant. Based on the widely accepted neuropsychological criteria for defining cognitive impairment^51,52^, participants with an RBANS total scale score of 1.5 s.d. below the normative mean or less were considered cognitively impaired. In the secondary care cohort, RBANS was not available. Instead, an RBANS proxy variable was created, based on similar paper-and-pencil neuropsychological tests (Supplementary Methods and Supplementary Table 18). The RBANS proxy was used as outcome in the secondary care cohort.

The secondary outcome ‘clinical AD’ was defined as having cognitive impairment (as described above) where AD was determined as the primary etiology. The latter was determined in consensus discussions including dementia specialists and neuropsychologists. The consensus discussions included access to medical examination by a dementia specialist at the memory clinic, a computed tomography or magnetic resonance imaging scan of the brain, CSF AD biomarkers or Aβ-PET results as well as neuropsychological assessment (Supplementary Methods). The diagnosis of clinical AD was defined in accordance with criteria from the International Working Group^13^, which includes a typical clinical presentation of AD in patients with objective cognitive impairment and confirmation with AD biomarkers. Participants fulfilling the criteria for clinical AD but also having signs of other concomitant diseases (for example, AD mixed with vascular disease) were labeled as having clinical AD in the analyses. Individuals that were negative according to this outcome were either cognitively unimpaired or cognitively impaired not due to AD (see Supplementary Table 16 for details of comorbidities and diagnoses).

The CDR scale is a semiobjective, qualitative staging instrument for the assessment dementia severity^32^. It assesses the cognitive and functional performance of everyday activities in six categories: memory, orientation, judgment and problem-solving, community affairs, home and hobbies, as well as personal affairs. Each item in the respective subscales is rated and a global CDR score is derived. A score of 0 is considered normal, with a score of 0.5, 1, 2 or 3 indicating questionable, mild, moderate and severe dementia^33^. Participants with a CDR global score of 0.5 and above were considered cognitively impaired and this was used as an outcome in the sensitivity analysis. In this study, CDR was performed by a dementia specialist at the memory clinic blinded to BioCog.

### Statistical analysis

The analyses were performed in Python v.3.9. Multiple logistic regression models were created, and cutoffs were established in the secondary care cohort. Model performance was then evaluated in the same cohort, and more importantly, in the external and completely independent primary care cohort. To decrease the risk of overfitting when creating multiple logistic regression models, the number of input variables was reduced with recursive feature elimination based on the standardized logistic regression model coefficients. The best model was selected using the AIC, requiring a decrease of 2 or more in AIC to indicate a better model fit and justifying the addition of another input variable^53,54^. Model performance was evaluated from the ROC AUC. Cutoffs were established for the probabilities from the logistic regression models. An optimal cutoff point was selected based on the maximum value of Youden’s index and the corresponding model was evaluated on accuracy, PPV, NPV, specificity and sensitivity. For the two-cutoff approach, cutoffs were selected based on 95% specificity and 95% sensitivity in the independent secondary care cohort. Participants in the intermediate group were not considered when evaluating model performance. The 95% CI and two-sided *P* values were computed using bootstrapping (*n* = 5,000 resamples with replacement), with a *P* value <0.05 indicating statistical significance. *P* values were adjusted for multiple comparisons by the Benjamini–Hochberg method. Differences between cognitively unimpaired and cognitively impaired on the various subtests of BioCog were assessed using the Mann–Whitney *U*-test. Spearman’s rank correlation coefficients were used to evaluate associations between BioCog subtests and their paper-and-pencil counterparts, as well as with age and education. Internal consistency was assessed using McDonald’s *ω*.

### Reporting summary

Further information on research design is available in the Nature Portfolio Reporting Summary linked to this article.

## Online content

Any methods, additional references, Nature Portfolio reporting summaries, source data, extended data, supplementary information, acknowledgements, peer review information; details of author contributions and competing interests; and statements of data and code availability are available at 10.1038/s41591-025-03965-4.

## Supplementary information

Supplementary Information Supplementary Methods, Figs. 1–8, Tables 1–18 and References.

Reporting Summary

## Data Availability

Anonymized data can be shared by request from qualified academic investigators for the purpose of replicating procedures and results presented in the article. Data transfer is required to be in agreement with EU legislation on the general data protection regulation and decisions by the Ethical Review Board of Sweden and Region Skåne, which should be regulated in a material transfer agreement.

## Code availability

No custom code or mathematical algorithm that was central to the conclusions was used in this study.
