# Supplementary material for: Primary care detection of Alzheimer’s disease using a self-administered digital cognitive test and blood biomarkers
Source: Nat Med. 2025 Sep 15;31(12):4131–9. doi: 10.1038/s41591-025-03965-4 (PMC12705462; doi:10.1038/s41591-025-03965-4)
Supplement: Supplementary file 1 — Supplementary Methods, Figs. 1–8, Tables 1–18 and References. [file 41591_2025_3965_MOESM1_ESM.pdf]

nature medicine

Supplementary information

https://doi.org/10.1038/s41591-025-03965-4

Primary care detection of Alzheimer's disease using a self-administered digital cognitive test and blood biomarkers

In the format provided by the authors and unedited

Supplementary Information

Supplementary Methods

Secondary care cohort: BioFINDER-2
Primary care cohort: BioFINDER primary care
Amyloid-β CSF and PET biomarkers
Comorbidities
RBANS proxy variable
Statistical analyses

Supplementary Tables

Supplementary Table 1. Spearman's correlations or Mann-Whitney U test between BioCog^{TM} and demographic variables: age, education level and sex in the secondary care cohort.
Supplementary Table 2. Spearman's correlations or Mann-Whitney U test between BioCog^{TM} and demographic variables: age, education level and sex in the primary care cohort.
Supplementary Table 3. Spearman's correlations between BioCog^{TM} and paper-and-pencil test scores in the secondary care cohort.
Supplementary Table 4. Spearman's correlations between BioCog^{TM} and paper-and-pencil test scores in the primary care cohort.
Supplementary Table 5. Mann-Whitney U test of the different BioCog^{TM} subtests between cognitively unimpaired and cognitively impaired in the secondary care cohort.
Supplementary Table 6. Mann-Whitney U test of the different BioCog^{TM} subtests between cognitively unimpaired and cognitively impaired in the primary care cohort.
Supplementary Table 7. Internal consistency for BioCog^{TM} assessed with McDonald´s omega in secondary and primary cohorts.
Supplementary Table 8. Completion times in minutes for BioCog^{TM} BioFINDER Primary Care cohort.
Supplementary Table 9. Input variables in the BioCog model development.
Supplementary Table 10. Head-to-head comparison between $BioCog_5$ (excluding age from $BioCog_6$) and other cognitive tests/composites when predicting the binary RBANS composite in the primary care cohort.
Supplementary Table 11. Significance comparison of the different workflows in primary care from Fig. 5.
Supplementary Table 12. Comparing a digital testing and blood biomarker based diagnostic workflow to standard paper-and-pencil tests and blood biomarkers to identify clinical AD.
Supplementary Table 13. Head-to-head comparison between $BioCog_6$ and other cognitive tests/composites when predicting CDR global score in the primary care cohort.
Supplementary Table 14. Extended metrics head-to-head comparison between $BioCog_6$ and other cognitive tests/composites when predicting the binary RBANS composite in the primary care cohort.
Supplementary Table 15. Extended metrics comparing a digital testing and blood biomarker based diagnostic workflow to the current standard clinical evaluation in the primary care cohort.
Supplementary Table 16. Underlying etiology to the cognitive impairment.
Supplementary Table 17. Characteristics of the subset of individuals with all existing data available for the BioCog^{TM} and blood biomarker workflow.
Supplementary Table 18: Description of the paper-and-pencil cognitive test variables used to predict a cognitive composite corresponding to an RBANS composite.

Supplementary Figures

Supplementary Figure 1. Flowchart**.** Enrollment flowchart
Supplementary Figure 2. Graphical interface of BioCog
Supplementary Figure 3. Self-reported BioCog experience questionnaire results.
Supplementary Figure 4. Head-to-head comparison between $BioCog_{PC}$, $BioCog_6$ and other cognitive tests/composites adjusted for demographic variables age, sex and education level.
Supplementary Figure 5. Comparing diagnostic workflows in the primary care cohort.

Supplementary Figure 6. Comparing a digital testing and blood biomarker based diagnostic workflow to the current standard clinical evaluation in the primary care cohort with an APS2 cutoff of 47.5.
Supplementary Figure 7. Evaluating models in the primary care cohort using CDR global score ≥ 0.5 as the reference standard for cognitive impairment.
Supplementary Figure 8. Establishing and evaluating a paper-and-pencil test based cognitive RBANS composite proxy variable in the primary care cohort.

References

Supplementary Methods

Secondary care cohort: BioFINDER-2

The Swedish prospective BioFINDER-2 study (NCT03174938) has been previously described.[1] The ongoing study consecutively enrolls participants at the Memory Clinic of Skåne University Hospital and the Memory Clinic of Ängelholm Hospital and comprises a diverse population of study participants. Besides cognitively unimpaired controls, it includes patients with cognitive symptoms subsequently categorized as subjective cognitive decline (SCD), mild cognitive impairment (MCI) or Alzheimer's disease (AD) with dementia as well as patients with other neurodegenerative diseases. For participants with SCD or MCI the inclusion criteria are: i) ages 40-100 years; ii) referred to the memory clinics due to cognitive symptoms; iii) MMSE score of 24-30 points; iv) does not fulfill the criteria for any dementia (major neurocognitive disorder) according to DSM-5, v) fluent in Swedish. Participants were classified as having MCI if they performed < -1.5 SD in any cognitive domain according to age and education stratified test norms. The neuropsychological battery covered the domains attention (Trail Making Test A and Symbol Digit Modalities Test), executive function (Trail Making Test B and A Quick Test of cognitive speed [AQT]), verbal ability (verbal fluency animals and the 15 word short version of the Boston Naming Test), memory (immediate and delayed recall from the Alzheimer's Disease Assessment Scale [ADAS]), and visuospatial function (incomplete letters and cube analysis from the Visual Object and Space Perception battery [VOSP]). Participants not classified as MCI were considered to have SCD. For participants with dementia due to AD the inclusion criteria are: i) ages 40-100 years; ii) referred to the memory clinics due to cognitive symptoms; iii) MMSE score of ≥12 points; iv) fulfill the *Diagnostic and Statistical Manual of Mental Disorders* [Fifth Edition] AD criteria for dementia (major neurocognitive disorder) due to Alzheimer disease and an abnormal Aβ-status (according to clinically used cutoff for CSF Aβ42/Aβ40 ratio, 0.072); v) fluent in Swedish. For participants with other non-AD dementias and neurodegenerative disorders the inclusion criteria are: i) ages 40-100 years; ii) fulfillment of criteria for dementia (major

neurocognitive disorder) due to frontotemporal dementia, Parkinson's disease (PD) with dementia, or subcortical vascular dementia, PD, progressive supranuclear palsy, multiple system atrophy, corticobasal syndrome or semantic variant primary progressive aphasia; and iii) fluent in Swedish. Exclusion criteria for all sub-cohorts are: i) significant unstable systemic illness that makes it difficult to participate in the study; ii) current significant alcohol or substance misuse; iii) refusing lumbar puncture, MRI or PET. For the present study, patients with either SCD, MCI or dementia were included. Participants performed the self-administered digital cognitive test battery, BioCog™, in conjunction to either their baseline or follow up MRI visit. Data was collected from October 2022 until December 2024.

Primary care cohort: BioFINDER primary care

The ongoing BioFINDER Primary-care study (NCT06120361) has been described earlier.[2] The inclusion criteria for the study are i) patient seeks primary care due to cognitive symptoms experienced by the patient and/or reported by an informant. Alternatively, when the primary care physician (PCP), after seeing the patient, suspects a progressive neurodegenerative disorder including, but not limited to, AD, Lewy body disease, frontotemporal lobar degeneration or subcortical vascular cognitive impairment; ii) age ≥40 years; and iii) cognitive impairment characterized as subjective cognitive decline, mild cognitive impairment or mild dementia. The exclusion criteria are i) already diagnosed with dementia; ii) significant unstable systemic illness making it difficult to participate in the study; iii) current significant alcohol or substance misuse; iv) refusing investigation at the Memory clinic; v) cognitive impairment with acute onset due to stroke; and vi) the cognitive impairment, as assessed by the PCP, can with high certainty be explained by another condition or disease such as psychotic disorder, depression or alcohol abuse. At their respective primary care center, patients underwent standard clinical evaluation ("standard-of-care"), including medical examination by the PCP, cognitive testing, standard blood assessments to rule out other causes and structural brain imaging. For this study

patients from 19 primary care centers were included and the study was integrated into routine clinical practice, therefore no extra personnel were hired at the primary care centers to conduct the study.

Amyloid-β CSF and PET biomarkers

CSF Aβ42/40 levels were analyzed using the Lumipulse assay (Fujirebio), which is clinically approved.[3] An abnormal Aβ42/40 ratio was defined as ≤0.072, in accordance with the FDA-cleared threshold and the reference value provided by the Clinical Chemistry Laboratory at Sahlgrenska University Hospital, Mölndal, Sweden. [18F]flutemetamol PET imaging was performed in LIST mode using GE Discovery MI scanners, between 90 and 110 minutes following intravenous administration of approximately 185 MBq of the tracer. A low-dose CT scan was conducted prior to PET acquisition for attenuation correction. PET images were attenuation corrected and reconstructed into four 5-minute frames using VPFX-S (ordered subset expectation maximization with time-of-flight and point spread function corrections), employing 6 iterations and 17 subsets. The reconstruction was carried out within a 25.6 cm field of view on a 256x256 matrix, yielding 1 mm^3 voxels. A 3 mm FWHM Gaussian filter was applied transaxially. Image interpretation (positive/negative) followed standardized visual reading guidelines by a single evaluator, with the exception that striatal retention was not assessed.

Comorbidities

Cardiovascular disease was defined as diagnosed hypertension, current anti-hypertensive/cardioprotective drugs or ischemic heart disease; hyperlipidemia as diagnosed hyperlipidemia; chronic kidney disease as an estimated glomerular filtration rate <60 ml/min/1.73 m2; and diabetes as diabetes type 1 or type 2.

RBANS proxy variable

As the RBANS test battery was not included in the secondary care cohort, we created a proxy variable based on 13 variables from very similar paper-and-pencil cognitive tests (Supplementary Table 18). In the primary care cohort, we created a multivariate logistic regression model that, after variable selection (same approach as for the models in main methods and results section), predicted the true binary RBANS variable. The best model included six variables (Delayed 10-word recall [correct answers], Symbol digit [correct answers], MMSE score [correct answers], Trail Making Test A [total time], Animal fluency [number correct], and Recognition test score [correct "no"-answers]), and demonstrated an AUC of 0.96 (Supplementary Figure 8). We used this model to create the RBANS proxy variable for each case in the secondary care cohort from the paper-and-pencil cognitive tests.

Statistical analysis

Standardized median differences were calculated as the difference between the medians divided by the pooled standard deviation. Standardized median differences and standardized differences in proportions 95% CI were calculated using 1000 bootstrap samples. Associations between BioCog and paper-and-pencil tests, age and education level were performed using Spearman´s rank correlations. Comparisons between BioCog scores by sex and cognitive status were performed using Mann-Whitney U test statistics.

Supplementary Tables

Supplementary Table 1. Spearman's correlations or Mann-Whitney U test between BioCog^{TM} and demographic variables: age, education level and sex in the secondary care cohort.

Measure	BioCog Immediate	BioCog Delayed	BioCog Recognition	BioCog Processing Speed
Age	-0.305<br>95%CI -0.423- -0.177<br>P = 4×10^{-5}	-0.457<br>95%CI -0.558- -0.343<br>P = 6×10^{-13}	-0.314<br>95%CI -0.431- -0.186<br>P = 2×10^{-6}	-0.578<br>95%CI -0.661- -0.480<br>P = 2×10^{-12}
Education	0.158<br>95%CI 0.023- 0.287<br>P = 0.018	0.158<br>95%CI 0.024- 0.287<br>P = 0.018	0.143<br>95%CI 0.008- 0.273<br>P = 0.033	0.128<br>95%CI -0.007- 0.259<br>ns
Sex (male)	Z= -0.465<br>ns<br>median=22<br>median=23	Z= -0.77<br>ns<br>median=3<br>median=3	Z= -0.197<br>ns<br>median=8<br>median=7	Z= -0.122<br>ns<br>median=33<br>median=34

Spearman´s rank correlation coefficients and Mann-Whitney U test (n=223).

Supplementary Table 2. Spearman's correlations or Mann-Whitney U test between BioCog^{TM} and demographic variables: age, education level and sex in the primary care cohort.

Measure	BioCog Immediate	BioCog Delayed	BioCog Recognition	BioCog Processing Speed
Age	-0.327<br>95%CI -0.414- -0.233<br>P = 1×10^{-11}	-0.400<br>95%CI -0.481- -0.312<br>P = 6×10^{-17}	-0.338<br>95%CI -0.424- -0.246<br>P = 3×10^{-12}	-0.557<br>95%CI -0.623- -0.484<br>P = 3×10^{-14}
Education	0.249<br>95%CI 0.152- 0.341<br>P = 4×10^{-7}	0.261<br>95%CI 0.165- 0.352<br>P = 1×10^{-7}	0.277<br>95%CI 0.182- 0.368<br>P = 1×10^{-8}	0.330<br>95%CI 0.238- 0.417<br>P = 1×10^{-11}
Sex (male)	Z= -2.566<br>P = 0.01<br>median=23<br>median=21	Z= -1.864<br>ns<br>median=2<br>median=2	Z= -2.243<br>P = 0.025<br>median=8<br>median=7	Z= -0.251<br>ns<br>median=32<br>median=32

Spearman´s rank correlation coefficients and Mann-Whitney U test (n=403).

Supplementary Table 3. Spearman's correlations between BioCog™ and paper-and-pencil test scores in the secondary care cohort.

Measure	BioCog Immediate	BioCog Delayed	BioCog Recognition	BioCog Processing Speed
ADAS wordlist immediate	0.658<br>95%CI 0.573-0.728<br>$P = 5 \times 10^{-29}$	0.684<br>95%CI 0.604-0.750<br>$P = 4 \times 10^{-32}$	0.619<br>95%CI 0.528-0.696<br>$P = 5 \times 10^{-25}$	0.599<br>95%CI 0.505-0.679<br>$P = 4 \times 10^{-23}$
ADAS wordlist Delayed	0.737<br>95%CI 0.668-0.793<br>$P = 1 \times 10^{-30}$	0.788<br>95%CI 0.730-0.834<br>$P = 2 \times 10^{-48}$	0.717<br>95% CI 0.645-0.777<br>$P = 1 \times 10^{-36}$	0.545<br>95%CI 0.443-0.634<br>$P = 1 \times 10^{-18}$
ADAS wordlist Recognition	0.680<br>95%CI 0.600-0.746<br>$P = 1 \times 10^{-31}$	0.647<br>95%CI 0.561-0.719<br>$P = 8 \times 10^{-28}$	0.612<br>95%CI 0.520-0.690<br>$P = 2 \times 10^{-24}$	0.431<br>95%CI 0.315-0.535<br>$P = 1 \times 10^{-11}$
Symbol Digit Modalities Test	0.554<br>95%CI 0.453-0.641<br>$P = 2 \times 10^{-19}$	0.527<br>95%CI 0.421-0.618<br>$P = 2 \times 10^{-17}$	0.454<br>95%CI 0.339-0.555<br>$P = 1 \times 10^{-12}$	0.786<br>95%CI 0.728-0.833<br>$P = 5 \times 10^{-44}$
VOSP* Incomplete Letters	0.159<br>95%CI 0.028-0.285<br>P = 0.018	0.132<br>95%CI 0.000-0.260<br>P = 0.05	0.135<br>95%CI 0.003-0.262<br>P =0.045	0.269<br>95%CI 0.142-0.387<br>$P = 5 \times 10^{-5}$

Spearman's rank correlation coefficients (n=223)
Abbreviations: ADAS, Alzheimer Disease Assessment Scale; CI, confidence interval; VOSP, Visual Object and Space Perception Battery.
*VOSP Incomplete Letters were available for 221 participants, cutoff as previously defined [4].

Supplementary Table 4. Spearman's correlations between BioCog™ and paper-and-pencil test scores in the primary care cohort.

Measure	BioCog Immediate	BioCog Delayed	BioCog Recognition	BioCog Processing Speed
ADAS wordlist immediate	0.622<br>95%CI 0.556-0.680<br>$P = 4 \times 10^{-44}$	0.632<br>95%CI 0.567-0.689<br>$P = 6 \times 10^{-46}$	0.562<br>95%CI 0.488-0.627<br>$P = 1 \times 10^{-34}$	0.557<br>95%CI 0.483-0.623<br>$P = 7 \times 10^{-34}$
ADAS wordlist Delayed	0.690<br>95%CI 0.634-0.740<br>$P = 1 \times 10^{-47}$	0.742<br>95%CI 0.7693-0.784<br>$P = 6 \times 10^{-41}$	0.683<br>95%CI 0.625-0.733<br>$P = 3 \times 10^{-44}$	0.578<br>95%CI 0.506-0.641<br>$P = 5 \times 10^{-37}$
ADAS wordlist Recognition	0.634<br>95%CI 0.570-0.691<br>$P = 3 \times 10^{-46}$	0.563<br>95%CI 0.489-0.628<br>$P = 1 \times 10^{-34}$	0.572<br>95%CI 0.500-0.636<br>$P = 3 \times 10^{-36}$	0.368<br>95%CI 0.278-0.453<br>$P = 2 \times 10^{-14}$
Symbol Digit Modalities Test	0.481<br>95%CI 0.400-0.556<br>$P = 4 \times 10^{-24}$	0.512<br>95%CI 0.433-0.583<br>$P = 1 \times 10^{-27}$	0.456<br>95%CI 0.372-0.533<br>$P = 8 \times 10^{-22}$	0.833<br>95%CI 0.793-0.857<br>$P = 2 \times 10^{-101}$
Digit Span Forward	0.108<br>95%CI 0.007-0.207<br>P = 0.033	0.109<br>95%CI 0.008-0.208<br>P = 0.030	0.148<br>95%CI 0.047-0.245<br>P = 0.003	0.289<br>95%CI 0.194-0.379<br>$P = 3 \times 10^{-9}$

Spearman's rank correlation coefficients were calculated on a subset of individuals with available data for all paper-and-pencil tests (n=397).
Abbreviations: ADAS, Alzheimer Disease Assessment Scale; CI, confidence interval.

Supplementary Table 5. Mann-Whitney U test of the different BioCog[TM] subtests between cognitively unimpaired and cognitively impaired in the secondary care cohort.

Measure	MMSE	BioCog Immediate	BioCog Delayed	BioCog Recognition	BioCog Processing Speed	Biocog
CU/CI	Z= -10.307<br>$P = 6 \times 10^{-35}$<br>median=29<br>median=24	Z= -9.846<br>$P = 7 \times 10^{-23}$<br>median=26<br>median=19	Z= -10.672<br>$P = 1 \times 10^{-26}$<br>median=5<br>median=1	Z= -9.426<br>$P = 4 \times 10^{-21}$<br>median=9<br>median=6	Z= -9.567<br>$P = 1 \times 10^{-21}$<br>median=43<br>median=27	Z= -11.738<br>$P = 8 \times 10^{-32}$<br>median=0.048<br>median=0.948

N=223 (104 cognitively unimpaired, 119 cognitively impaired)
Abbreviations: CU, Cognitively Unimpaired; CI, Cognitively Impaired; MMSE, Mini-Mental State Examination.

Supplementary Table 6. Mann-Whitney U test of the different BioCog^{TM} subtests between cognitively unimpaired and cognitively impaired in the primary care cohort.

Measure	MMSE*	BioCog Immediate	BioCog Delayed	BioCog Recognition	BioCog Processing Speed	$BioCog_6$ probability
CU/CI	Z= -11.674<br>$P = 1 \times 10^{-31}$<br>median=29<br>median=25	Z= -12.457<br>$P = 1 \times 10^{-35}$<br>median=25<br>median=19	Z= -13.085<br>$P = 4 \times 10^{-39}$<br>median=4<br>median=1	Z= -12.075<br>$P = 1 \times 10^{-33}$<br>median=9<br>median=6	Z= -12.615<br>$P = 1 \times 10^{-36}$<br>median=43<br>median=26	Z= -14.674<br>$P = 9 \times 10^{-49}$<br>median=0.183<br>median=0.962

N=403 (174 cognitively unimpaired, 229 cognitively impaired)
Abbreviations: CU, Cognitively Unimpaired; CI, Cognitively Impaired; MMSE, Mini-Mental State Examination.
* MMSE existed for 402/403 participants

Supplementary Table 7. Internal consistency for BioCog^{TM} assessed with McDonald´s omega in secondary and primary cohorts.

Secondary Care cohort	McDonald´s Omega
BioCog Immediate recall (3 items)	0.873
BioCog Immediate recall total time (3 items)	0.704
BioCog Processing Speed (4 items)	0.906
Primary Care cohort	
BioCog Immediate recall (3 items)	0.865
BioCog Immediate recall Time (3 items)	0.773
BioCog Processing Speed (4 items)	0.974

Supplementary Table 8. Completion times in minutes for BioCog BioFINDER Primary Care cohort.

CI 229	m=11.5, sd=1.05
CU 174	m=10.9, sd=0.89
All participants	m=11.2, sd=1.02

Supplementary Table 9. Input variables in the BioCog model development.

BioCog model subtest or demographic variable	Variable type/unit	Used in $BioCog_6$ model
Delayed 10-word recall	Number of correct answers	Yes
Delayed 10-word recognition	Number of correct answers	Yes
Cognitive processing speed task	Number of correct answers	Yes
Three repetitions of immediate 10-word recall	Number of correct answers	Yes
Three repetitions of immediate 10-word recall	Total time	Yes
Orientation to time	Number of correct answers	No
Age	Years	Yes
Sex	Male/Female	No
Education level	Years	No

Supplementary Table 10. Head-to-head comparison between $BioCog_5$ (excluding age from $BioCog_6$) and other cognitive tests/composites when predicting the binary RBANS composite in the primary care cohort.

	Cutoff(s) for positivity	Accuracy (95% CI, FDR corrected P-value compared against $BioCog_5$)	PPV (95% CI, FDR corrected P-value compared against $BioCog_5$)	NPV (95% CI, FDR corrected P-value compared against $BioCog_5$)	Specificity (95% CI, FDR corrected P-value compared against $BioCog_5$)	Sensitivity (95% CI, FDR corrected P-value compared against $BioCog_5$)	Intermediate (95% CI, FDR corrected P-value compared against $BioCog_5$)
**One-cutoff approaches**							
**$BioCog_5$**	>0.551	86% (83%-90%)	87% (83%-92%)	85% (80%-90%)	84% (78%-89%)	88% (84%-92%)	-
**MMSE**	<27	71% (67%-76%, 0.0007)	81% (75%-87%, 0.02)	64% (58%-70%, 0.0007)	80% (75%-86%, 0.3)	64% (58%-71%, 0.0007)	-
**MoCA**	<26	67% (62%-71%, 0.0007)	63% (58%-68%, 0.0007)	93% (86%-100%, 0.06)	27% (20%-34%, 0.0007)	98% (96%-100%, 0.0007)	-
**Mini-Cog**	<4	75% (71%-79%, 0.0007)	78% (72%-83%, 0.0007)	71% (64%-78%, 0.0007)	72% (65%-79%, 0.003)	77% (71%-83%, 0.0007)	-
**CANTAB**	>41	76% (71%-80%, 0.0007)	78% (73%-83%, 0.001)	73% (66%-79%, 0.0007)	72% (65%-79%, 0.006)	78% (73%-84%, 0.0007)	-
**Two-cutoff approaches**							
**$BioCog_5$**	>0.857 = positive <0.376 = negative	93% (91%-96%)	97% (94%-99%)	90% (85%-95%)	96% (93%-99%)	91% (86%-95%)	26% (22%-31%)
**MoCA**	<24 = positive >26 = negative	76% (71%-81%, 0.001)	74% (68%-79%, 0.001)	90% (80%-100%, 0.9)	31% (22%-40%, 0.001)	98% (96%-100%, 0.003)	24% (20%-28%, 0.5)

Comparisons were made on a subset of individuals with available data for all cognitive tests (n=381). All cutoffs were based on previous literature or established in the secondary care cohort. Confidence intervals and two-sided P-values were computed using bootstrapping.

Abbreviations: CANTAB, Cambridge Neuropsychological Test Automated Battery; CI, Confidence Interval; FDR, False Discovery Rate; MMSE, Mini-Mental State Examination; MoCA, Montreal Cognitive Assessment.

Supplementary Table 11. Significance comparison of the different workflows in primary care from Fig. 5.

**Evaluation metric**	**Accuracy**			
Workflow	BioCog$_6$ & APS2	BioCog$_6$ & APS2 (two-cutoff)	PCP only	APS2 only
BioCog$_6$ & APS2	-	P = 0.0006	P = 0.0006	P = 0.0006
BioCog$_6$ & APS2 (two-cutoff)	P = 0.0006	-	P = 0.0006	P = 0.0006
PCP only	P = 0.0006	P = 0.0006	-	P = 0.0006
APS2 only	P = 0.0006	P = 0.0006	P = 0.0006	-
**Evaluation metric**	**PPV**			
Workflow	BioCog$_6$ & APS2	BioCog$_6$ & APS2 (two-cutoff)	PCP only	APS2 only
BioCog$_6$ & APS2	-	P = 0.0006	P = 0.0006	P = 0.0006
BioCog$_6$ & APS2 (two-cutoff)	P = 0.0006	-	P = 0.0006	P = 0.0006
PCP only	P = 0.0006	P = 0.0006	-	P = 0.01
APS2 only	P = 0.0006	P = 0.0006	P = 0.01	-
**Evaluation metric**	**NPV**			
Workflow	BioCog$_6$ & APS2	BioCog$_6$ & APS2 (two-cutoff)	PCP only	APS2 only
BioCog$_6$ & APS2	-	P = 0.01	P = 0.0006	P = 0.02
BioCog$_6$ & APS2 (two-cutoff)	P = 0.01	-	P = 0.0006	P = 0.8
PCP only	P = 0.0006	P = 0.0006	-	P = 0.0006
APS2 only	P = 0.02	P = 0.8	P = 0.0006	-
**Evaluation metric**	**Specificity**			
Workflow	BioCog$_6$ & APS2	BioCog$_6$ & APS2 (two-cutoff)	PCP only	APS2 only
BioCog$_6$ & APS2	-	P = 0.003	P = 0.0006	P = 0.0006
BioCog$_6$ & APS2 (two-cutoff)	P = 0.003	-	P = 0.0006	P = 0.0006
PCP only	P = 0.0006	P = 0.0006	-	0.7
APS2 only	P = 0.0006	P = 0.0006	0.7	-
**Evaluation metric**	**Sensitivity**			
Workflow	BioCog$_6$ & APS2	BioCog$_6$ & APS2 (two-cutoff)	PCP only	APS2 only
BioCog$_6$ & APS2	-	P = 0.004	P = 0.0006	P = 0.0006
BioCog$_6$ & APS2 (two-cutoff)	P = 0.004	-	P = 0.0006	P = 0.9
PCP only	P = 0.0006	P = 0.0006	-	P = 0.0006
APS2 only	P = 0.0006	P = 0.9	P = 0.0006	-

Comparisons were made on a subset of individuals with all existing data available (n=365). Two-sided P-values were computed using bootstrapping (n=5000 resamples with replacement), with a P-value less than 0.05 indicating statistical significance. P-values were adjusted for multiple comparisons by the Benjamini–Hochberg method.
Abbreviations: APS, Amyloid Probability Score-2; PCP, Primary Care Physician.

Supplementary Table 12. Comparing a digital testing and blood biomarker based diagnostic workflow to standard paper-and-pencil tests and blood biomarkers to identify clinical AD.

	**Cutoff(s) for positivity**	**Accuracy** (95% CI, FDR corrected P-value compared against $BioCog_6$ & APS2)	**PPV** (95% CI, FDR corrected P-value compared against $BioCog_6$ & APS2)	**NPV** (95% CI, FDR corrected P-value compared against $BioCog_6$ & APS2)	**Specificity** (95% CI, FDR corrected P-value compared against $BioCog_6$ & APS2)	**Sensitivity** (95% CI, FDR corrected P-value compared against $BioCog_6$ & APS2)
**$BioCog_6$ & APS2**	>0.575<br>>36	90% (86%-93%)	83% (76%-89%)	93% (89%-96%)	92% (89%-95%)	84% (77%-91%)
**MMSE & APS2**	<27<br>>36	84% (80%-87%, 0.0006)	80% (71%-88%, 0.4)	85% (80%-89%, 0.0006)	92% (89%-96%, 0.9)	64% (55%-73%. 0.0006)
**MoCA & APS2**	<26<br>>36	83% (79%-87%, 0.0006)	67% (59%-74%, 0.0006)	95% (91%-98%, 0.1)	79% (74%-84%, 0.0006)	90% (84%-95%, 0.04)
**APS2**	>36	80% (76%-84%, 0.0006)	63% (55%-70%, 0.0006)	95% (92%-98%, 0.02)	75% (69%-80%, 0.0006)	92% (87%-97%, 0.0006)

Comparisons were made on a subset of individuals in the primary care cohort with all existing data available (n=365). All cutoffs were based on previous literature or established in the secondary care cohort. Confidence intervals and two-sided P-values were computed using bootstrapping.
Abbreviations: APS, Amyloid Probability Score-2; MMSE, Mini-Mental State Examination; MoCA, Montreal Cognitive Assessment.

Supplementary Table 13. Head-to-head comparison between $BioCog_6$ and other cognitive tests/composites when predicting CDR global score in the primary care cohort.

	Cutoff(s) for positivity	Accuracy (95% CI, FDR corrected P-value compared against $BioCog_6$)	PPV (95% CI, FDR corrected P-value compared against $BioCog_6$)	NPV (95% CI, FDR corrected P-value compared against $BioCog_6$)	Specificity (95% CI, FDR corrected P-value compared against $BioCog_6$)	Sensitivity (95% CI, FDR corrected P-value compared against $BioCog_6$)	Intermediate (95% CI, FDR corrected P-value compared against $BioCog_6$)
**One-cutoff approaches**							
**$BioCog_6$**	>0.575	81% (76%-85%)	84% (79%-89%)	75% (68%-82%)	77% (69%-84%)	83% (78%-88%)	-
**MMSE**	<27	69% (64%-74%, 0.0008)	83% (77%-89%, 1.0)	58% (51%-65%, 0.0008)	81% (74%-87%, 0.4)	62% (55%-69%. 0.0008)	-
**MoCA**	<26	69% (65%-74%, 0.0008)	67% (62%-72%, 0.0008)	86% (75%-95%, 0.08)	28% (20%-36%, 0.0008)	97% (94%-99%, 0.0008)	-
**Mini-Cog**	<4	71% (66%-76%, 0.0008)	79% (73%-85%, 0.06)	62% (54%-70%, 0.0008)	71% (63%-78%, 0.2)	72% (66%-78%, 0.0008)	-
**CANTAB**	>41	73% (68%-78%, 0.007)	79% (73%-85%, 0.05)	65% (57%-72%, 0.009)	70% (62%-78%, 0.1)	75% (69%-81%, 0.01)	-
**Two-cutoff approaches**							
**$BioCog_6$**	>0.769 = positive <0.332 = negative	87% (83%-91%)	90% (86%-95%)	81% (74%-88%)	85% (77%-91%)	88% (83%-93%)	19% (15%-23%)
**MoCA**	<24 = positive >26 = negative	75% (70%-81%, 0.001)	75% (69%-80%, 0.001)	82% (66%-95%, 0.1)	29% (19%-39%, 0.001)	97% (94%-99%, 0.001)	24% (19%-28%, 0.2)

Comparisons were made on a subset of individuals with existing data for all cognitive tests (n=344). All cutoffs were based on previous literature or established in the secondary care cohort. Confidence intervals and two-sided P-values were computed using bootstrapping.

Abbreviations: CANTAB, Cambridge Neuropsychological Test Automated Battery; CI, Confidence Interval; FDR, False Discovery Rate; MMSE, Mini-Mental State Examination; MoCA, Montreal Cognitive Assessment.

Supplementary Table 14. Extended metrics head-to-head comparison between BioCog$_6$ and other cognitive tests/composites when predicting the binary RBANS composite in the primary care cohort.

	Cutoff(s) for positivity	Balanced accuracy (95% CI, FDR corrected P-value compared against BioCog$_6$)	F1-score (95% CI, FDR corrected P-value compared against BioCog$_6$)	Positive likelihood ratio (95% CI, FDR corrected P-value compared against BioCog$_6$)	Negative likelihood ratio (95% CI, FDR corrected P-value compared against BioCog$_6$)
**BioCog$_6$**	>0.575	84% (80%-87%)	86% (83%-90%)	4.52 (3.34-6.27)	0.14 (0.09-0.20)
**MMSE**	<27	72% (68%-77%, 0.0006)	72% (66%-77%, 0.0006)	3.50 (2.54-5.01, 0.3)	0.44 (0.35-0.52, 0.0006)
**MoCA**	<26	63% (59%-66%, 0.0006)	77% (73%-80%, 0.0006)	1.36 (1.25-1.50, 0.0006)	0.05 (0.00-0.12, 0.05)
**Mini-Cog**	<4	75% (70%-79%, 0.0006)	77% (73%-82%, 0.0006)	2.88 (2.25-3.75, 0.02)	0.31 (0.23-0.40, 0.0006)
**CANTAB**	>41	75% (71%-79%, 0.003)	78% (74%-82%, 0.0006)	2.93 (2.28-3.79, 0.02)	0.29 (0.21-0.38, 0.001)
**BioCog$_6$**	>0.769 = positive <0.332 = negative	89% (86%-93%)	91% (88%-94%)	7.45 (4.80-12.30)	0.08 (0.04-0.13)
**MoCA**	<24 = positive >26 = negative	64% (60%-69%, 0.0006)	84% (80%-88%, 0.0006)	1.44 (1.26-1.66, 0.0006)	0.05 (0.00-0.12, 0.4)

Comparisons were made on a subset of individuals with existing data for all cognitive tests (n=381). All cutoffs were based on previous literature or established in the secondary care cohort. Confidence intervals and two-sided P-values were computed using bootstrapping. Abbreviations: CANTAB, Cambridge Neuropsychological Test Automated Battery; CI, Confidence Interval; FDR, False Discovery Rate; MMSE, Mini-Mental State Examination; MoCA, Montreal Cognitive Assessment.

Supplementary Table 15. Extended metrics comparing a digital testing and blood biomarker based diagnostic workflow to the current standard clinical evaluation in the primary care cohort.

	Balanced accuracy (95% CI, FDR corrected P-value compared against $BioCog_6$ & APS2)	F1-score (95% CI, FDR corrected P-value compared against $BioCog_6$ & APS2)	Positive likelihood ratio (95% CI, FDR corrected P-value compared against $BioCog_6$ & APS2)	Negative likelihood ratio (95% CI, FDR corrected P-value compared against $BioCog_6$ & APS2)
**$BioCog_6$ & APS2**	88% (84%-91%)	83% (78%-88%)	11.1 (7.22-17.4)	0.17 (0.10-0.25)
**PCP_{AD}**	66% (61%-71%, 0.0006)	54% (46%-61%, 0.0006)	2.42 (1.83-3.19, 0.0006)	0.57 (0.44-0.70, 0.0006)
**APS2 only**	83% (79%-87%, 0.006)	74% (68%-80%, 0.0006)	3.71 (2.97-4.70, 0.0006)	0.11 (0.04-0.18, 0.01)

Comparisons were made on a subset of individuals with all existing data available (n=365). Confidence intervals and two-sided P-values were computed using bootstrapping.
Abbreviations: APS, Amyloid Probability Score-2; PCP, Primary Care Physician.

Supplementary Table 16. Underlying etiology to the cognitive impairment

Clinical diagnosis	
Cognitively unimpaired	174 (42%)
AD	75 (18.6%)
AD+VaD	45 (11.2%)
AD+DLB	5 (1.2%)
VaD	39 (9,7%)
CI NOS	34 (8.4%
Dementia NOS	13 (3.2)
DLB	5 (1.2%)
PSP/CBS	3 (0.07%)
Alcohol	2 (0.05%)
NPH	2 (0.05%)
Other disorders	2 (0.05%)
PDD	1 (0.02%)
Psychiatric disorder	1 (0.02%)
Traumatic Brain Injury	1 (0.02%)
svPPA	1 (0.02%)

All conditions listed above were at the cognitive impaired (CI) stage. CI was determined based on RBANS total index score >1.5 SD below normative age corrected mean.

Abbreviations: AD, Alzheimer's disease; CI, Cognitively Impaired; DLB, dementia with Lewy bodies (or prodromal DLB); NOS, not otherwise specified; NPH, normal pressure hydrocephalus (or hydrocephalus); PDD, Parkinson's disease dementia; PSP, progressive supranuclear palsy; svPPA, semantic variant primary progressive aphasia; VaD, vascular disease/dementia.

Supplementary Table 17. Characteristics of the subset of individuals with all existing data available for the BioCog^{TM} and blood biomarker workflow.

	Primary care cohort
**No. of participants**	365
**Age median (IQR), years**	77 (72 to 82)
**Sex, No. (%)**	
Female	188 (52)
Male	177 (48)
**Length of education median (IQR), years**	11 (9 to 13)
**Carrier of apolipoprotein E ε4 No./total (%)**	151/364 (41)
**Mini-Mental State Examination**	
No. of participants	364
Score, median (IQR)	27 (25 to 29)
**Cognitive impairment (RBANS<78) No. (%)**	
Negative	154 (42)
Positive without dementia	115 (32)
Positive with dementia	96 (26)
**Amyloid-β status[a] No./total (%)**	
Negative	175 (48)
Positive	190 (52)
**Plasma Amyloid probability score 2[b]**	25 (8 to 82)
**Cognitively impaired due to Alzheimer's disease[c] No./total (%)**	
Negative	250 (68)
Positive	115 (32)
**Medical history No. (%)**	
Cardiovascular disease	274 (75)
Hyperlipidemia	225 (62)
Chronic kidney disease	96 (26)
Diabetes	72 (20)

[a] Biomarker confirmed, based on either CSF Aβ42/Aβ40 or Aβ-PET.

[b] The percentage of p-tau217 divided by non-phosphorylated-tau217 multiplied by 100 and combined with the Aβ42:Aβ40 plasma ratio into a predefined logistic regression model using a predefined cutoff optimized for the clinical practice study (Palmqvist et al. JAMA).

[c] Based on a consensus diagnosis including CSF analysis or PET done by dementia experts.

Supplementary Table 18: Description of the paper-and-pencil cognitive test variables used to predict a cognitive composite corresponding to an RBANS composite.

Paper-and-pencil test	Description
Symbol digit	Number of correct answers in a substitution task. Based on a reference key, the test taker has 90 seconds to pair numbers and geometric figures.
Immediate 10-word recall failure 1	Number of words not immediately recalled from a 10-word list (first attempt).
Immediate 10-word recall failure 2	Number of words not immediately recalled from a 10-word list (second attempt).
Immediate 10-word recall failure 3	Number of words not immediately recalled from a 10-word list (third attempt).
Immediate 10-word recall average	Number of words not immediately recalled from a 10-word list (average from three attempts).
Delayed 10-word recall	Number of words not recalled after a delay from a 10-word list.
Recognition test "yes/no"	Number of correctly recognized words from a 10-word list.
Animal fluency	Number of animals the test taker can name during 60 seconds of time.
Trail Making Test A	The time it takes for the test taker to draw a line to connect 25 consecutive numbers.
Trail Making Test B	The time it takes for the test taker to draw a line alternating between 13 consecutive numbers and 12 consecutive letters.
Mini-Mental State Examination	Number of correct answers in the mini-mental-state-examination (maximum score=30). The test consists of 20 questions divided into 11 areas covering orientation to time and location, memory, language and visuospatial functions.
Letter S fluency	Number of words that starts with the letter "s" the test taker can name during 60 seconds of time.

Supplementary Figures

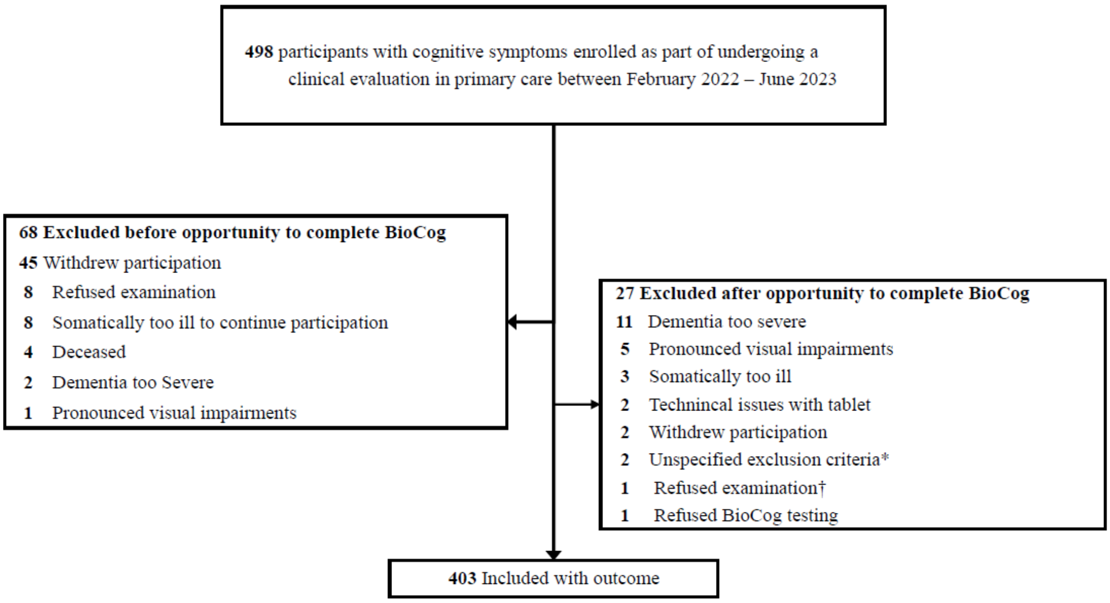

Supplementary Figure 1. Enrollment Flowchart. Flowchart shows exclusion after enrollment. The definition for enrollment was signing of the informed consent.
* Did not fulfill study criteria but exact criterion for exclusion was not available.
† Refused lumbar puncture and Aβ-PET.

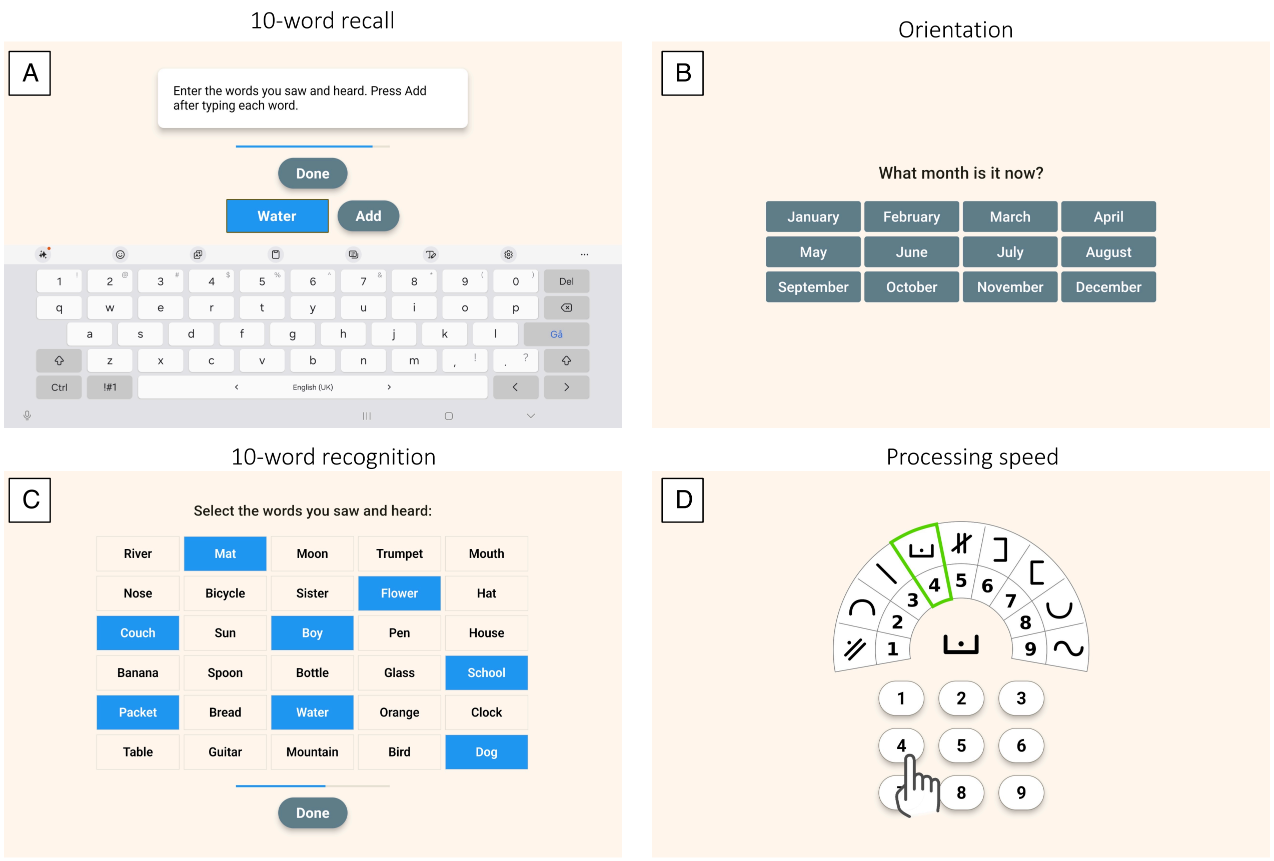

Supplementary Figure 2. Graphical interface of BioCog™.
Example of the BioCog™ sub-tests performed on a tablet. A) 10-word recall task. B) Orientation to the month. C) 10-word recognition. D) Cognitive processing speed.

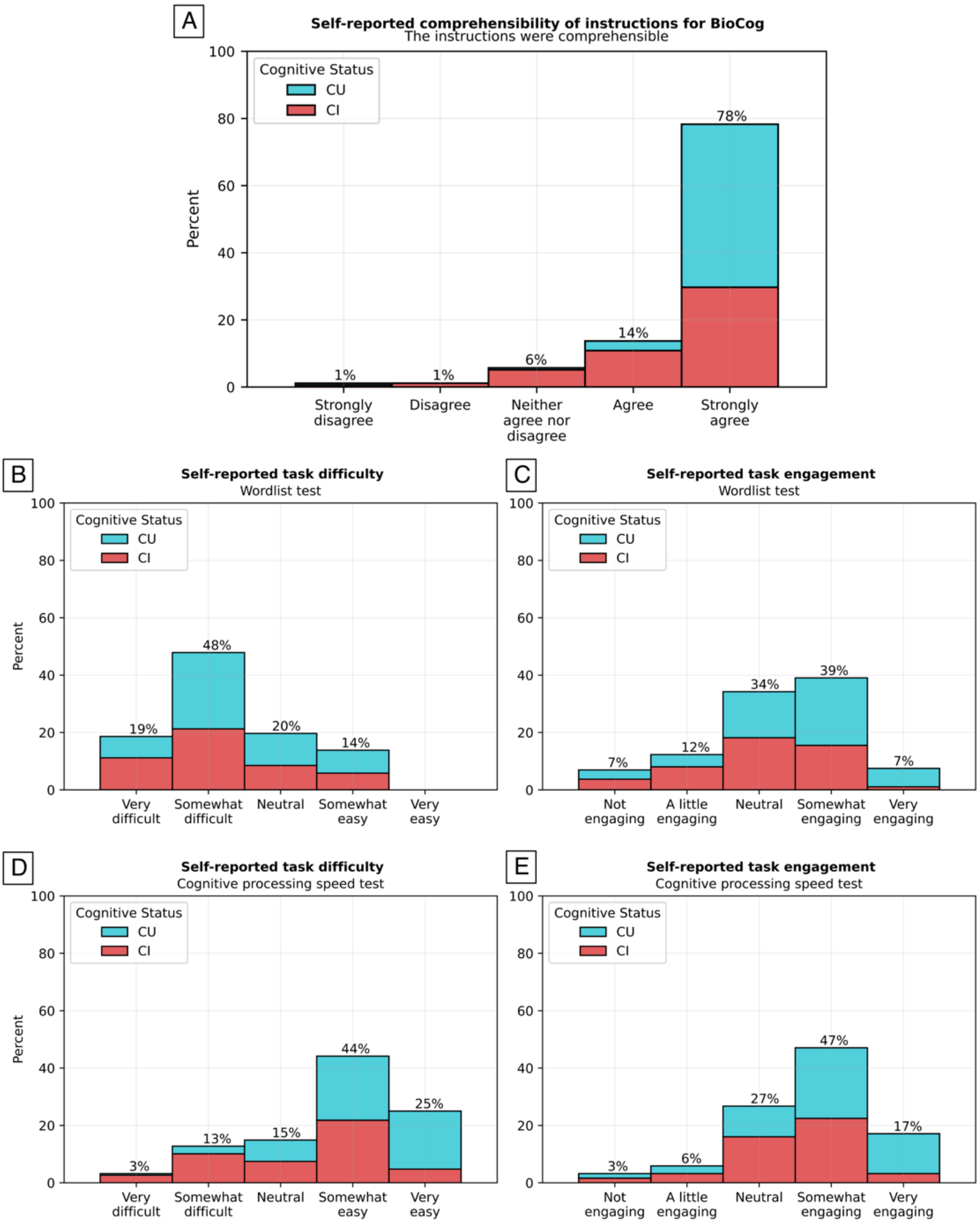

Supplementary Figure 3. Self-reported BioCog experience questionnaire results.

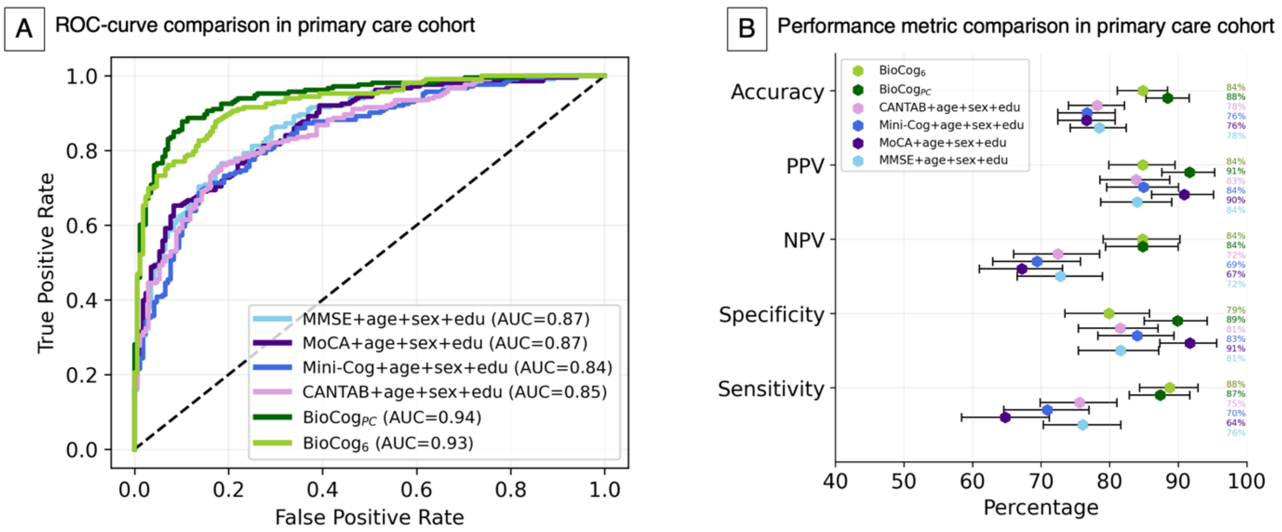

Supplementary Figure 4. Head-to-head comparison between BioCog$_{PC}$, BioCog$_6$ and other cognitive tests/composites adjusted for demographic variables age, sex and education level. Logistic regression model performances in the primary care cohort (which was not used to fit the BioCog$_6$ model but used to fit all other models). This comparison potentially overestimates the performance of the cognitive test/composite + demographic models relative to BioCog$_6$, as only BioCog$_6$ was evaluated on unseen data. Comparisons were made on a subset of individuals with existing data for all cognitive tests/composites and demographic variables (n=381). The numeric cognitive test/composites scores (treated as continuous variables) were used as input. Error bars represent 95% confidence intervals, calculated using bootstrapping.

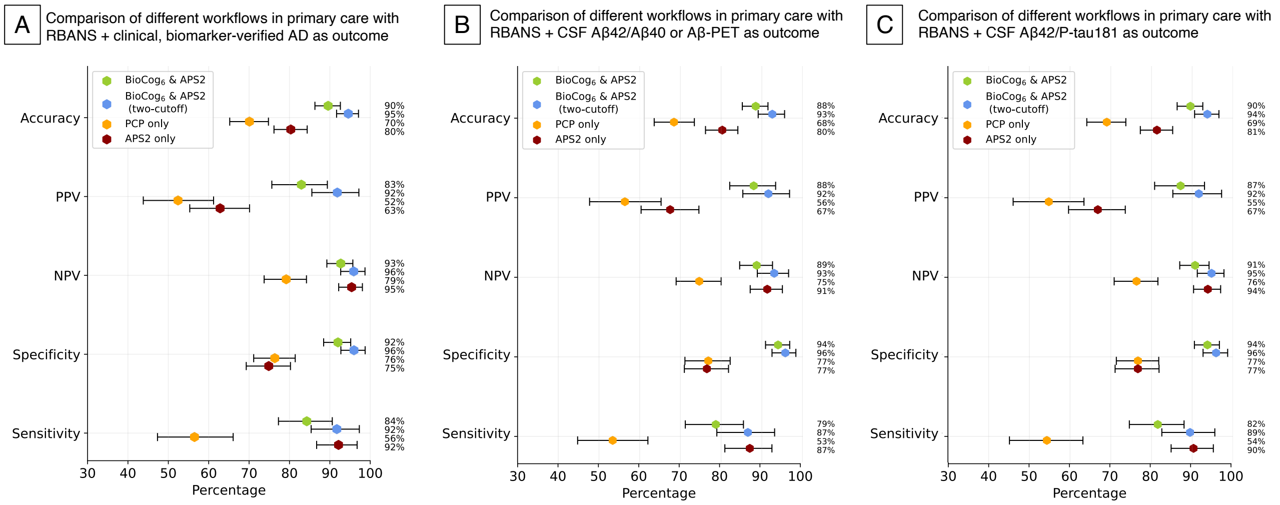

Supplementary Figure 5. Comparing diagnostic workflows in the primary care cohort.
Comparisons were made on a subset of individuals with all existing data available (n=365). Evaluation of the workflow using our $BioCog_6$ model for step 1 and the plasma biomarker APS2 for step 2 with a one-cutoff (green) or two-cutoff (blue) approach. For the two-cutoff approach, two cutoffs were applied both for $BioCog_6$ and plasma APS2. The workflow was compared against a standard clinical evaluation by primary care physicians without any biomarkers (orange) and using only the plasma biomarker APS2 without any cognitive assessment (red). A) Same as Fig. 5b but also with the two-cutoff approach. B) Same as A) but with cognitive impairment + CSF Aβ42/Aβ40 or Aβ-PET as outcome. C) Same as A) but with cognitive impairment + CSF Aβ42/P-tau181 as outcome.

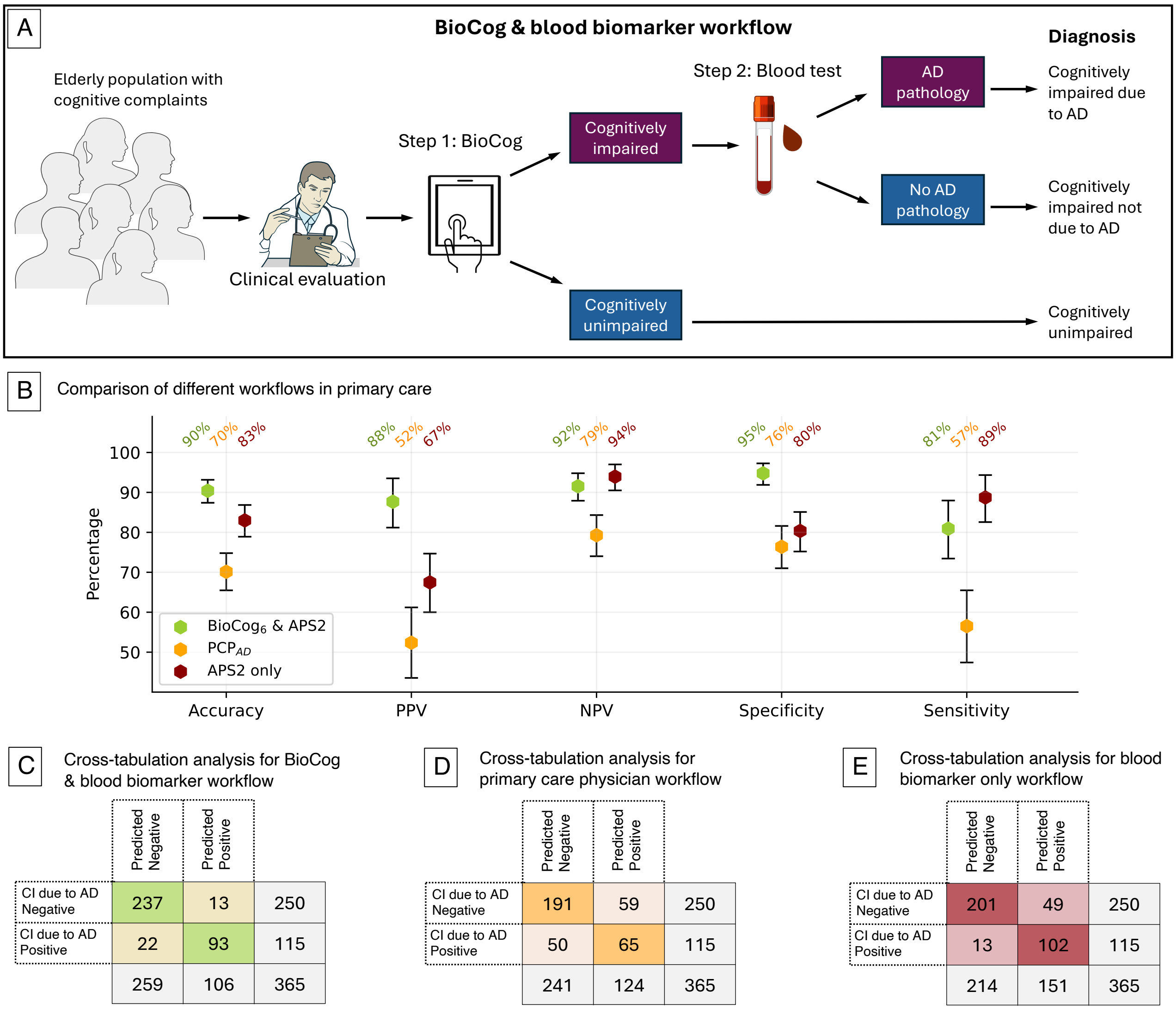

Supplementary Figure 6. Comparing a digital testing and blood biomarker based diagnostic workflow to the current standard clinical evaluation in the primary care cohort with an APS2 cutoff of 47.5.[5] Comparisons were made on a subset of individuals with all existing data available (n=365, see Supplementary Tab. 7 for detailed population characteristics). A) Our proposed primary care two-step workflow consisting of step 1) detection of cognitive impairment using the BioCog™ followed by step 2) in the cognitively impaired individuals, a blood biomarker assessment to evaluate if AD pathology is present. B) Evaluation of the workflow using our $BioCog_6$ model for step 1 and the plasma biomarker APS2 for step 2 (green). The workflow was compared against a standard clinical evaluation by primary care physicians where the physician assesses both whether the patient had cognitive impairment (MCI or dementia) and if it was caused by AD (without any biomarkers) (PCP_{AD}, orange), and against a workflow using only the plasma biomarker APS2 without any cognitive assessment (red). C)-E) Corresponding cross-tabulation analyses. Illustrations from NIAID NIH BIOART Source, bioart.niaid.nih.gov.

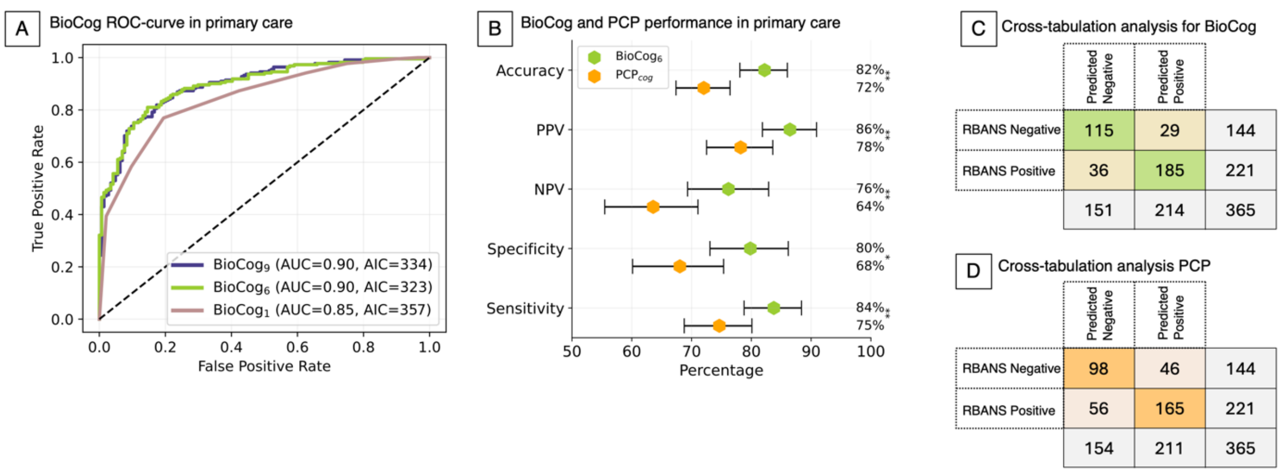

Supplementary Figure 7. Evaluating models in the primary care cohort using CDR global score ≥ 0.5 as the reference standard for cognitive impairment. Model performances in the primary care cohort (which was not used to fit the models nor to establish the cutoff). A) ROC-curves, AUCs and AICs for the three BioCog™ models using 1, 6 or 9 input variables. B) Evaluation of $BioCog_6$ based on the optimal cutoff point established in the secondary care cohort (probability=0.575). Compared against a PCP diagnosis of SCD versus MCI/dementia (PCP_{cog}). Error bars indicate 95% CIs, with the centre point corresponding to the mean value. C) Cross-tabulation analysis using $BioCog_6$. D) Cross-tabulation analysis using PCP_{cog} diagnoses. All analyses were made on a subset of individuals with existing CDR and PCP_{cog} evaluation data (n=365).
*$P < 0.05$, **$P < 0.01$, ***$P < 0.001$ (assessed with bootstrapping and FDR corrected).
Exact P-values in B): $P_{Accuracy} = 0.004$, $P_{PPV} = 0.003$, $P_{NPV} = 0.003$, $P_{Specificity} = 0.02$, $P_{Sensitivity} = 0.004$.

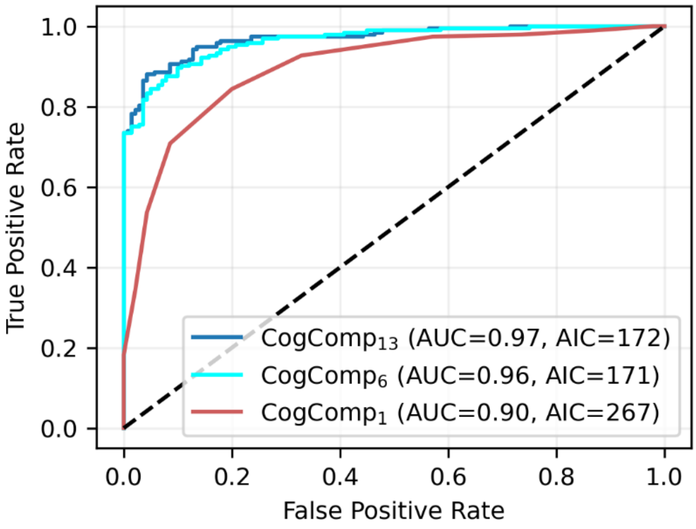

Supplementary Figure 8. Establishing and evaluating a paper-and-pencil test based cognitive RBANS composite proxy variable in the primary care cohort. ROC-curves, AUCs and AICs for the three cognitive composite models using 1, 6 or 13 input variables.

References

1. Palmqvist, S., *et al.* Discriminative Accuracy of Plasma Phospho-tau217 for Alzheimer Disease vs Other Neurodegenerative Disorders. *JAMA* **324**, 772-781 (2020).
2. Palmqvist, S., *et al.* Blood biomarkers to detect Alzheimer disease in primary care and secondary care. *JAMA* (2024).
3. US Food and Drug Administration. Evaluation of automatic class III designation for Lumipulse G β-Amyloid Ratio (1-42/1-40): decision summary. **2025**.
4. Quadalti, C., *et al.* Clinical effects of Lewy body pathology in cognitively impaired individuals. *Nature medicine* **29**, 1964-1970 (2023).
5. Meyer, M.R., *et al.* Clinical validation of the PrecivityAD2 blood test: A mass spectrometry-based test with algorithm combining %p-tau217 and Abeta42/40 ratio to identify presence of brain amyloid. *Alzheimers Dement* **20**, 3179-3192 (2024).
