# Supplementary material for: Primary care detection of Alzheimer’s disease using a self-administered digital cognitive test and blood biomarkers
Source: Nat Med. 2025 Sep 15;31(12):4131–9. doi: 10.1038/s41591-025-03965-4 (PMC12705462; doi:10.1038/s41591-025-03965-4)
Supplement: Supplementary file 2 — Reporting Summary [file 41591_2025_3965_MOESM2_ESM.pdf]

nature portfolio

Corresponding author(s): Oskar Hansson

Last updated by author(s): Jul 19, 2025

Reporting Summary

Nature Portfolio wishes to improve the reproducibility of the work that we publish. This form provides structure for consistency and transparency in reporting. For further information on Nature Portfolio policies, see our Editorial Policies and the Editorial Policy Checklist.

Statistics

For all statistical analyses, confirm that the following items are present in the figure legend, table legend, main text, or Methods section.

n/a	Confirmed	
☐	☒	The exact sample size (*n*) for each experimental group/condition, given as a discrete number and unit of measurement
☐	☒	A statement on whether measurements were taken from distinct samples or whether the same sample was measured repeatedly
☐	☒	The statistical test(s) used AND whether they are one- or two-sided<br>*Only common tests should be described solely by name; describe more complex techniques in the Methods section.*
☐	☒	A description of all covariates tested
☐	☒	A description of any assumptions or corrections, such as tests of normality and adjustment for multiple comparisons
☐	☒	A full description of the statistical parameters including central tendency (e.g. means) or other basic estimates (e.g. regression coefficient) AND variation (e.g. standard deviation) or associated estimates of uncertainty (e.g. confidence intervals)
☐	☒	For null hypothesis testing, the test statistic (e.g. *F*, *t*, *r*) with confidence intervals, effect sizes, degrees of freedom and *P* value noted<br>*Give P values as exact values whenever suitable.*
☒	☐	For Bayesian analysis, information on the choice of priors and Markov chain Monte Carlo settings
☒	☐	For hierarchical and complex designs, identification of the appropriate level for tests and full reporting of outcomes
☐	☒	Estimates of effect sizes (e.g. Cohen's *d*, Pearson's *r*), indicating how they were calculated

Our web collection on statistics for biologists contains articles on many of the points above.

Software and code

Policy information about availability of computer code

Data collection	The BioCog Application for Andriod was used to collect data.
Data analysis	Python version 3.9 (packages: sklearn 0.0, pandas 1.4.4, numpy 1.23.3, matplotlib 3.5.3, statsmodels 0.13.2) and SPSS version 27.

For manuscripts utilizing custom algorithms or software that are central to the research but not yet described in published literature, software must be made available to editors and reviewers. We strongly encourage code deposition in a community repository (e.g. GitHub). See the Nature Portfolio guidelines for submitting code & software for further information.

Data

Policy information about availability of data

All manuscripts must include a data availability statement. This statement should provide the following information, where applicable:

- Accession codes, unique identifiers, or web links for publicly available datasets
- A description of any restrictions on data availability
- For clinical datasets or third party data, please ensure that the statement adheres to our policy

Anonymized data can be shared by request from qualified academic investigators for the purpose of replicating procedures and results presented in the article. Data transfer is required to be in agreement with EU legislation on the general data protection regulation and decisions by the Ethical Review Board of Sweden and Region Skåne.

Research involving human participants, their data, or biological material

Policy information about studies with human participants or human data. See also policy information about sex, gender (identity/presentation), and sexual orientation and race, ethnicity and racism.

Reporting on sex and gender	We used the term "sex" throughout the manuscript. Sex was determined based on self-reporting. Statistical analyses included sex as predictor or covariate; the study included two independent cohorts altogether comprised of balanced numbers of males (n=194 in the primary care cohort and 128 in the secondary care cohort) and females (n=209 in the primary care cohort and 95 in the secondary care cohort); therefore, we believe the findings apply to both sexes.
Reporting on race, ethnicity, or other socially relevant groupings	This study did not include categorization of race, ethnicity and/or other socially relevant groupings.
Population characteristics	We included 223 participants with cognitive symptoms from a secondary care cohort: the ongoing BioFINDER-2 study (NCT03174938). The mean (SD) age was 73 (8.8) years, 128 (57%) were male, and 119 (53%) had objectively verified cognitive impairment. Additionally, 403 participants were included from a primary care cohort, BioFINDER-Primary Care study (NCT06120361, Supplementary Fig. 1), which recruits patients seeking help for cognitive symptoms at 19 primary care units. Only patients for whom the primary care physician thought a neurodegenerative disease was reasonably possible to cause the symptomatology were offered to participate in the study. The mean (SD) age of the primary care cohort was 77 (8.0) years, 194 (48%) were male, and 229 (57%) had objectively verified cognitive impairment. Detailed information is given in Table 1.
Recruitment	Participants from two cohorts were included, one from secondary care and one from primary care. Data for this study were collected between February 2022 and December 2024.<br><br>Secondary care participants were recruited from the ongoing BioFINDER-2 study (NCT03174938). Briefly, the study consecutively enrolls participants at the secondary care Memory Clinic of Skåne University Hospital and the Memory Clinic of Ängelholm Hospital and includes a diverse population of study participants.<br><br>The BioFINDER-Primary Care study (NCT06120361), is an ongoing study that recruits patients from primary care centers in southern Sweden. The study consecutively includes patients seeking medical help due to cognitive symptoms either self-reported or reported by a close relative or spouse. Additionally, inclusion may also be initiated based on the primary care physician's suspicion of a neurocognitive disorder.
Ethics oversight	All participants provided written informed consent prior to enrollment in the study, and ethical approval for the study was obtained from the Swedish Ethical Review Authority.

Note that full information on the approval of the study protocol must also be provided in the manuscript.

Field-specific reporting

Please select the one below that is the best fit for your research. If you are not sure, read the appropriate sections before making your selection.

☒ Life sciences ☐ Behavioural & social sciences ☐ Ecological, evolutionary & environmental sciences

For a reference copy of the document with all sections, see nature.com/documents/nr-reporting-summary-flat.pdf

Life sciences study design

All studies must disclose on these points even when the disclosure is negative.

Sample size	We included all available participants with complete data from from the secondary care cohort (n=223) and the primary care cohort (n=403). Following the commonly cited rule of thumb for logistic regression analyses of a minimum of 10 events per predictor variable (Peduzzi, P. et al. 1996), our sample satisfied this criterion in all models.
Data exclusions	Data were limited to participants that had performed BioCog and had complete data for each analysis. See flowchart Supplementary Figure 1 for detailed exclusion after enrollment.
Replication	To develop a robust BioCog model and reduce the risk of overfitting, we applied recursive feature selection with AIC penalization and focused on external model validation in the independent primary care cohort. Performance metrics included bootstrapped confidence intervals to quantify uncertainty and support the reliability of the findings.
Randomization	In these two prospective cohort studies (observational studies) no allocation into experimental groups were performed, therefore randomization is not relevant to this study. Statistical analyses were controlled for potential confounding effects of age, sex and education.
Blinding	CSF, plasma and PET analyses were performed by individuals blinded to all clinical data. RBANS was performed by neuropsychologists and CDR was performed by dementia specialists, all at the memory clinic blinded to BioCog.

Reporting for specific materials, systems and methods

We require information from authors about some types of materials, experimental systems and methods used in many studies. Here, indicate whether each material, system or method listed is relevant to your study. If you are not sure if a list item applies to your research, read the appropriate section before selecting a response.

Materials & experimental systems

n/a	Involved in the study
☒	☐ Antibodies
☒	☐ Eukaryotic cell lines
☒	☐ Palaeontology and archaeology
☒	☐ Animals and other organisms
☐	☒ Clinical data
☒	☐ Dual use research of concern
☒	☐ Plants

Methods

n/a	Involved in the study
☒	☐ ChIP-seq
☒	☐ Flow cytometry
☒	☐ MRI-based neuroimaging

Clinical data

Policy information about clinical studies

All manuscripts should comply with the ICMJE guidelines for publication of clinical research and a completed CONSORT checklist must be included with all submissions.

Clinical trial registration

BioFINDER-2 study: NCT03174938. BioFINDER-Primary Care study: NCT06120361.

Study protocol

BioFINDER-2: https://clinicaltrials.gov/ct2/show/NCT03174938
BioFINDER-Primary Care study: https://clinicaltrials.gov/ct2/show/NCT06120361

Data collection

Data for this study were collected between February 2022 and December 2024.

Secondary care participants were recruited from the ongoing BioFINDER-2 study (NCT03174938). Briefly, the study consecutively enrolls participants at the secondary care Memory Clinic of Skåne University Hospital and the Memory Clinic of Ängelholm Hospital and includes a diverse population of study participants.

The BioFINDER-Primary Care study (NCT06120361), is an ongoing study that recruits patients from primary care centers in southern Sweden. The study consecutively includes patients seeking medical help due to cognitive symptoms either self-reported or reported by a close relative or spouse. Additionally, inclusion may also be initiated based on the primary care physician's suspicion of a neurocognitive disorder

Outcomes

The primary outcome was objectively verified cognitive impairment, established using RBANS (primary care) or an RBANS proxy variable (secondary care).
The secondary outcome "clinical AD" was defined as having cognitive impairment where AD was determined as the primary etiology.
In a sensitivity analysis, CDR was used to define objectively verified cognitive impairment.

Plants

Seed stocks

Report on the source of all seed stocks or other plant material used. If applicable, state the seed stock centre and catalogue number. If plant specimens were collected from the field, describe the collection location, date and sampling procedures.

Novel plant genotypes

Describe the methods by which all novel plant genotypes were produced. This includes those generated by transgenic approaches, gene editing, chemical/radiation-based mutagenesis and hybridization. For transgenic lines, describe the transformation method, the number of independent lines analyzed and the generation upon which experiments were performed. For gene-edited lines, describe the editor used, the endogenous sequence targeted for editing, the targeting guide RNA sequence (if applicable) and how the editor was applied.

Authentication

Describe any authentication procedures for each seed stock used or novel genotype generated. Describe any experiments used to assess the effect of a mutation and, where applicable, how potential secondary effects (e.g. second site T-DNA insertions, mosiacism, off-target gene editing) were examined.
